# Nobiletin fortifies mitochondrial respiration in skeletal muscle to promote healthy aging against metabolic challenge

**DOI:** 10.1038/s41467-019-11926-y

**Published:** 2019-08-28

**Authors:** Kazunari Nohara, Venkata Mallampalli, Travis Nemkov, Marvin Wirianto, Jiah Yang, Youqiong Ye, Yuxiang Sun, Leng Han, Karyn A. Esser, Eugenia Mileykovskaya, Angelo D’Alessandro, Carla B. Green, Joseph S. Takahashi, William Dowhan, Seung-Hee Yoo, Zheng Chen

**Affiliations:** 1 Department of Biochemistry and Molecular Biology, The University of Texas Health Science Center at Houston, 6431 Fannin Street, Houston, Texas 77030 USA; 2 Department of Biochemistry and Molecular Genetics, University of Colorado Denver - Anschutz Medical Campus, Aurora, Colorado 80045 USA; 3 Department of Nutrition and Food Science, Texas A&M University, College Station, Texas 77843 USA; 4 Department of Physiology and Functional Genomics, University of Florida College of Medicine, Gainesville, Florida 32610-0274 USA; 5 Department of Neuroscience, The University of Texas Southwestern Medical Center, 5323 Harry Hines Boulevard, Dallas, Texas 75390 USA; 6 Howard Hughes Medical Institute, The University of Texas Southwestern Medical Center, Dallas, Texas 75390 USA

**Keywords:** Biochemistry, Genetics, Physiology

## Abstract

Circadian disruption aggravates age-related decline and mortality. However, it remains unclear whether circadian enhancement can retard aging in mammals. We previously reported that the small molecule Nobiletin (NOB) activates ROR (retinoid acid receptor-related orphan receptor) nuclear receptors to potentiate circadian oscillation and protect against metabolic dysfunctions. Here we show that NOB significantly improves metabolic fitness in naturally aged mice fed with a regular diet (RD). Furthermore, NOB enhances healthy aging in mice fed with a high-fat diet (HF). In HF skeletal muscle, the NOB-ROR axis broadly activates genes for mitochondrial respiratory chain complexes (MRCs) and fortifies MRC activity and architecture, including Complex II activation and supercomplex formation. These mechanisms coordinately lead to a dichotomous mitochondrial optimization, namely increased ATP production and reduced ROS levels. Together, our study illustrates a focal mechanism by a clock-targeting pharmacological agent to optimize skeletal muscle mitochondrial respiration and promote healthy aging in metabolically stressed mammals.

## Abstract (web summary)

The small molecule Nobiletin enhances circadian rhythms and protects against obesity-associated metabolic dysfunction in mice. Here the authors test its effect on health and lifespan, reporting that circadian enhancement promotes fitness and healthy aging in metabolically challenged mice.

## Introduction

Metabolic deterioration is a hallmark of aging^1^. The rate of the metabolic syndrome, a cluster of metabolic risk factors including central obesity, glucose intolerance, insulin resistance, and dyslipidemia, is higher in the elderly than in young adults. Consistent with the observed energy imbalance where energy expenditure lags behind intake in the elderly, the body mass index increases during aging^2^. Such exaggerated adiposity negatively influences organ functions throughout the body including skeletal muscle, the largest, mitochondria-rich metabolic organ with crucial roles in activity, thermogenesis, and overall energy homeostasis. Increased intramuscular fat content can adversely influence energy homeostasis, in part attributable to progressive decline in mitochondrial function and ATP production in the skeletal muscle^1^. A dynamic and reciprocal interplay between mitochondria and aging involves gene regulation for mitochondrial components, mitochondrial dynamics, and reactive oxygen species (ROS)^1^. More recently, supercomplex (SC) organization of mitochondrial respiratory complexes (MRCs) has been postulated to facilitate electron transfer and gate radical escape^3,4^, improving ATP production while minimizing oxidative damage. Such MRC architecture has been implicated in aging^5^, although different studies have documented distinct alterations in MRC SCs in aged tissues^6,7^. Overall, it is important to understand mitochondrial functional regulation in aging.

The endogenous circadian clock is a fundamental regulatory mechanism for metabolism and aging. Cell-autonomous molecular oscillators contain positive (CLOCK, BMAL1, and RORs) and negative (CRYPTOCHROME1/2, PERIOD1-3, and REV-ERBs) functional components forming transcription–translation loops^8^. These oscillators regulate metabolism throughout the body^9,10^. For example, the core clock genes *Clock* and *Bmal1* were found to be essential for skeletal muscle microfilament architecture and force generation; interestingly, significant impairments in mitochondrial volume and respiratory function were observed in these circadian clock-deficient skeletal muscle^11^. Coincident with metabolic and physiological decline, aging is characterized by circadian dysfunction and attenuation^12,13^. Whereas expression of the core clock gene appear largely unaffected during aging^14^, the robustness of clock-regulated gene expression^14^ and physiological outputs, including suprachiasmatic nucleus (SCN) firing rate, secretion of metabolic regulatory hormones (e.g., cortisol and melatonin), thermogenesis, and sleep architecture, is impaired with age^12,15^. Moreover, circadian response to entraining cues in both animals and humans was found to be weaker and slower with age^16,17^. Importantly, circadian disruption in rodents, via genetic mutation or environmental perturbation, can accelerate aging and mortality^18,19^. For example, *Bmal1* knockout mice, known to suffer loss of behavioral rhythmicity and defective energy homeostasis, displayed early aging in multiple organs and shortened lifespan^20^.

Consistent with a functional role of circadian deterioration during aging^12,13^, emerging evidence supports a beneficial role of robust circadian rhythms in aging. Long-lived αMUPA transgenic mice sustained high-amplitude circadian rhythms even in older age^21^ and implantation of young SCN in aged hamsters increased amplitude and improved longevity^19^. In accordance, anti-aging dietary interventions provide initial clues that circadian enhancement may mediate, at least in part, the beneficial effects against age-related decline^14,22,23^. For example, whereas the obesogenic high-fat diet (HFD) increases the risk of metabolic disease and early mortality, and leads to dampened circadian gene expression^24^, time-restriction feeding (TRF), namely limiting daily HFD intake to a circadian time window of 12 h or less, markedly promotes energy expenditure and potentiates circadian gene oscillation in mice^25^, and decelerates cardiac aging in *Drosophila*^23^. The beneficial effects of TRF in *Drosophila* were found to involve circadian clocks and mitochondrial electron transport chain (ETC) complexes^23^. Likewise, caloric restriction (CR) has been shown to consolidate feeding within a narrow circadian window and augment circadian gene oscillation and metabolic rhythms including lipid flux^14,22,26–28^.

Various small molecules and/or dietary components (e.g., Resveratrol) have shown promising lifespan-extending effects^29,30^; however, it is unclear to what extent the clock plays a role in the process. In a complementary approach, a growing number of pharmacological agents have also been identified to directly target circadian clocks or clock components^31–33^. Given the strong correlation between aging and dampened clocks, we are interested in exploiting pharmacological agents to enhance aged clocks^34,35^. Recently, we identified a natural flavonoid called Nobiletin (NOB) that targets the ROR nuclear receptors in the secondary loop of the oscillator. Consistent with a critical role of RORs in circadian amplitude control, NOB was found to enhance circadian rhythms in cultured cells and mouse tissues, and strongly improved energy homeostasis in metabolic disease model mice^36^. In this study, we interrogate a potential anti-aging function of NOB in aged mice under both normal and nutritional excess conditions. Our results uncover a key role of this clock-enhancing agent to promote healthy aging under metabolic challenge via a concerted optimization of mitochondrial respiration. Therefore, this study establishes the importance of robust circadian functions for healthspan and illustrates cellular pathways amenable to pharmacological manipulation.

## Results

### NOB effects in aged mice with regular diet

**Fig. 1:**
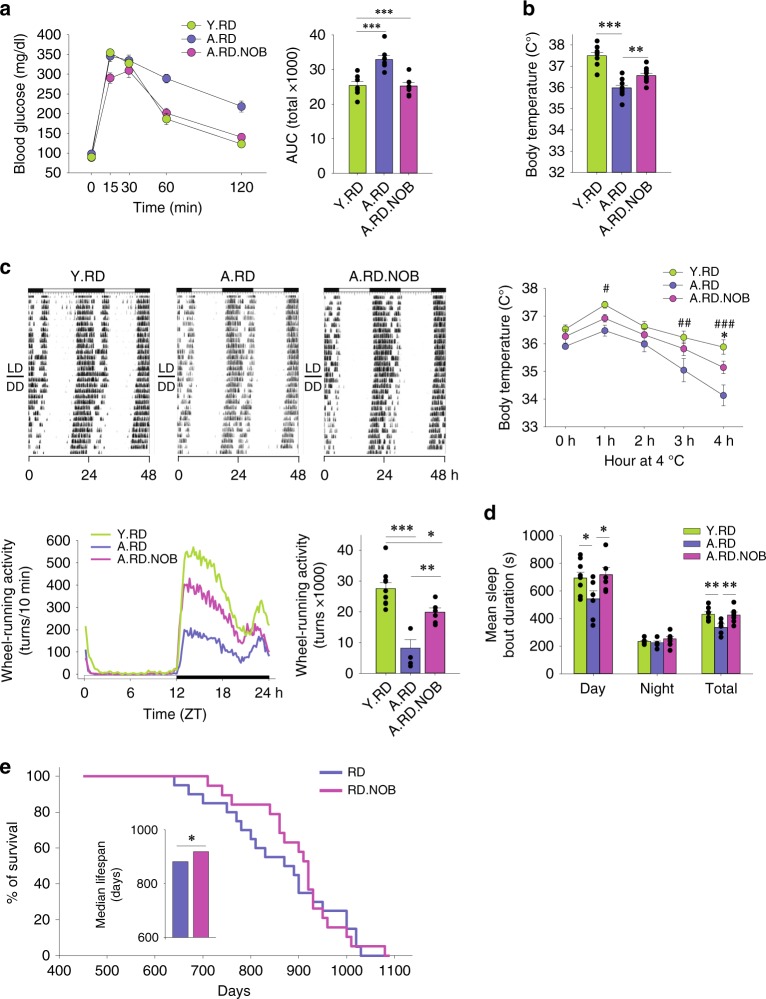
NOB improved physiological health and survival of aged mice under regular diet. **a**–**d** Young (Y) or aged (A) mice were fed with regular diet (RD) or regular diet with NOB (RD.N). **a** Glucose tolerance test and area under curve (AUC) (*n* = 10, 7, and 8 for Y.RD, A.RD, and A.RD.NOB, respectively). **b** Core body temperature (upper panel, *n* = 10) and cold tolerance (lower panel, *n* = 10, 7, and 5 for Y.RD, A.RD, and A.RD.NOB, respectively); *A.RD vs. A.RD.NOB; #A.RD vs. Y.RD; two-way ANOVA). **c** Wheel-running activity (WRA) analysis. Representative actograms (upper panels) are shown. Daily activity patterns (lower left, line graph) and daily averages (lower right, bar graph) are shown (ActiView) (*n* = 10, 5, and 6 for Y.RD, A.RD, and A.RD.NOB, respectively). **d** Mean sleep bout duration was analyzed with the PIEZO sleep analysis system (*n* = 9, 6, and 6 for Y.RD, A.RD, and A.RD.NOB, respectively). **e** Kaplan–Meier (KM) survival curves in 16-month-old mice fed with regular diet with or without 0.1% NOB supplement. Inset: median lifespan comparison. **a**–**d** *p < 0.05, **p < 0.01, ***p < 0.001, one-way ANOVA. **e** *p < 0.05, Log-rank and Mann–Whitney *U*-tests (see Supplementary Fig. 1h). Data are presented as mean ± SEM in bar and line graphs

Our previous study with young mice (2–4 months) revealed that NOB conferred robust metabolic protection against overnutrition, but showed little effect in a normal-fat regular diet (RD) condition^36,37^. To investigate NOB effects in aging and circadian rhythms, we first fed aged mice (20–22 months old at the beginning of the experiment) with a RD with or without 0.1% NOB for up to 20 weeks, with young (6-month-old) mice as a control. With RD feeding, NOB did not significantly change overall body weight, lean/fat mass, food intake, or respiratory quotient in aged mice (Supplementary Fig. 1a–c), similar to previous results in RD-fed young mice. However, although aged mice (A.RD) showed impaired glucose tolerance compared with young mice (Y.RD), NOB treatment (A.RD.NOB) fully restored glucose tolerance in aged mice (Fig. 1a), illustrating a corrective effect of NOB on glucose homeostasis during aging. Furthermore, in conjunction with a trend for increased heat production (energy expenditure) as measured in metabolic chamber (Supplementary Fig. 1d), NOB increased basal body temperature and cold tolerance in aged mice (Fig. 1b). Furthermore, circadian behavioral assays uncovered that NOB led to a twofold increase in distance run/day on voluntary wheels compared with aged RD-fed mice during the active phase (Fig. 1c). Collectively, although aging diminishes energy expenditure, NOB restored glucose homeostasis and promoted energy expenditure and circadian activity in aged mice.

Next, we investigated several aging markers. Treadmill assays showed no improvement between control and NOB groups (Supplementary Fig. 1e), indicating that although NOB increased voluntary running activity in circadian wheel-running assays, it did not improve forced exercise endurance. Similarly, NOB showed no effects on grip strength in aged mice (Supplementary Fig. 1f). We next conducted piezo noninvasive sleep assays to measure sleep. Although the total amount of sleep was not altered (Supplementary Fig. 1g), the mean sleep bout duration, a measure of sleep consolidation, is strongly elevated to levels seen in young mice (Fig. 1d). Finally, to investigate NOB effects on longevity, we performed survival studies where mice, starting at 16 months of age, were maintained under constant husbandry conditions and not subjected to experimental manipulation. Although NOB showed little effect on maximum lifespan (Fig. 1e), quartile analysis of the survival curves suggests enhancement in median lifespan compared with the control group (Fig. 1e, insert; Supplementary Fig. 1h, Log-rank and Mann–Whitney *U*-tests, *p* < 0.05), with NOB-treated mice living more than 1 month longer than control group at 50% death. These results collectively indicate beneficial effects of NOB on healthy aging and midlife survival in normal feeding conditions.

### NOB effects in aged mice fed with HFD

**Fig. 2:**
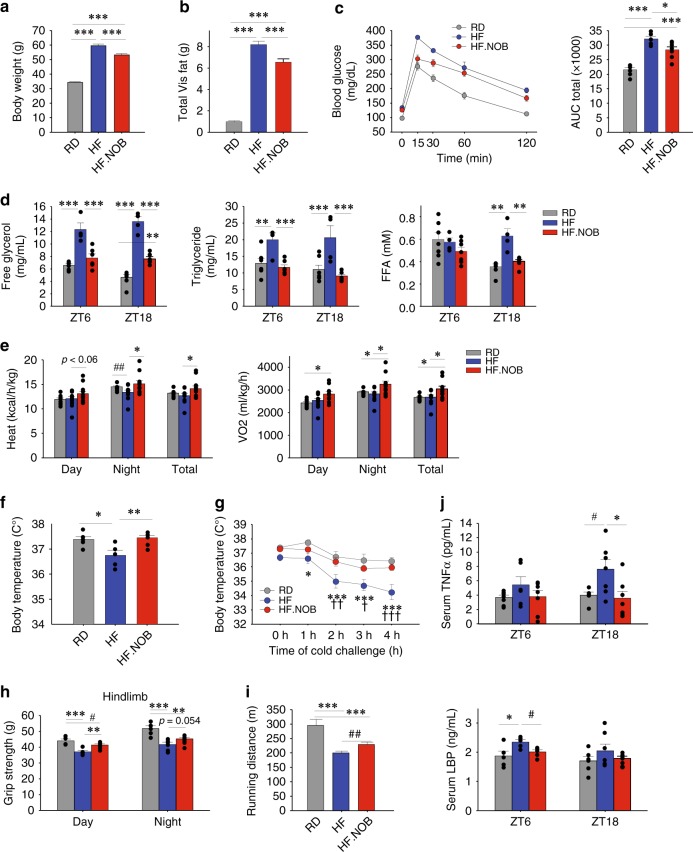
NOB improved adiposity, glucose homeostasis, and fitness in high-caloric conditions. Aged mice were fed with regular diet (RD), high-fat (HF) diet, or HF diet with NOB (HF.NOB). **a** End point (after 20–22 weeks of treatment) body weight and **b** total Visceral (Vis) fat mass (including perigonadal fat, perirenal fat, and mesenteric fat) were measured (*n* = 20, 16, and 17 for RD, HF, and HF.NOB, respectively). **c** Glucose tolerance test and area under curve (AUC) (*n* = 6, 5, and 7 for RD, HF, and HF.NOB, respectively). **d** Serum lipid homeostasis parameters were measured at indicated time points (*n* = 7, 4, and 6 for RD, HF, and HF.NOB, respectively). **e** In vivo heat production and oxygen consumption measured in metabolic chamber. Average values of day, night, and total are shown (*n* = 9, 10, and 10 for RD, HF, and HF.NOB, respectively). **f** Core body temperature (*n* = 6, 4, and 7 for RD, HF, and HF.NOB, respectively). **g** Cold tolerance test (*n* = 6, 5, and 7 for RD, HF, and HF.NOB, respectively; *HF vs. RD, †HF vs. HF.NOB, two-way ANOVA). **h** Hindlimb grip strength test (*n* = 5, 6, and 8 for RD, HF, and HF.NOB, respectively). **i** Running distances were measured with treadmill under HF conditions (*n* = 12, 11, and 12 for RD, HF, and HF.NOB, respectively). **j** Inflammation markers including TNFα (upper panel) and LBP (lower panel) (ZT6, *n* = 7, 6, and 7 for RD, HF, and HF.NOB, respectively; ZT18, *n* = 6, 8, and 7 for RD, HF, and HF.NOB, respectively). Color schemes are the same in all panels: gray, RD; blue, HF; red, HF.NOB. *p < 0.05, **p < 0.01, ***p < 0.001, one-way ANOVA; #*p* < 0.05, ##*p* < 0.01, ###*p* < 0.001, *t*-test. Data are presented as mean ± SEM in bar and line graphs

Given the robust effects of NOB against nutrient excess in young mice^36^, we next examined whether NOB can improve energy homeostasis in HFD-fed aged mice. As in young mice, the HF.NOB-fed aged mice showed reduced overall body weight and visceral, subcutaneous, and total fat pad weights relative to HFD-fed controls (Fig. 2a, b and Supplementary Fig. 2a, b), whereas the calorie intake was not significantly changed by NOB (Supplementary Fig. 2c). HF.NOB-fed mice showed improved glucose tolerance compared with the HF group (Fig. 2c), suggesting improved glucose homeostasis. To evaluate NOB effects on lipid homeostasis, serum lipids, including free glycerol, triglyceride (TG), and free fatty acid (FFA), were measured at ZT6 and ZT18 (Zeitgeber Time). Compared with RD, HFD feeding led to marked increases in serum lipids, whereas HF.NOB feeding strongly reversed the HF induction (Fig. 2d).

We next examined in vivo energy homeostasis by metabolic chamber. The respiratory quotient remained largely unaltered by NOB (Supplementary Fig. 2d). In contrast, although HF reduced heat production relative to RD, NOB treatment strongly increased heat production and oxygen consumption, particularly during the dark/active phase (Fig. 2e and Supplementary Fig. 2e). Likewise, whereas HF feeding reduced core body temperature and cold tolerance compared with RD, NOB fully restored these parameters to normal levels (Fig. 2f, g). In accordance, NOB ameliorated the dramatic increase in brown adipose tissue (BAT) weight in HFD feeding and also upregulated *Ucp1* expression (Supplementary Fig. 2f), further indicating a role of NOB against age-related decline in energy expenditure and adaptive thermogenesis. In Piezo sleep assays, NOB did not restore sleep bout duration in HFD feeding compared with RD (Supplementary Fig. 2g). On the other hand, although HFD-fed mice showed much attenuated hindlimb grip strength and exercise tolerance, NOB treatment partially yet significantly rescued these fitness parameters in HF.NOB mice (Fig. 2, i and Supplementary Fig. 2h). Finally, we examined inflammation markers in the serum. NOB significantly ameliorated HF-induced increases in the levels of tumor necrosis factor-α (TNFα) and lipopolysaccharide-binding peptide (LBP) (Fig. 2j), with lesser effects on interleukin (IL)-6 (Supplementary Fig. 2i). Together, these results indicate a robust efficacy of NOB to enhance various healthy aging parameters.

### ROR-NOB regulates behavior and muscle gene expression

**Fig. 3:**
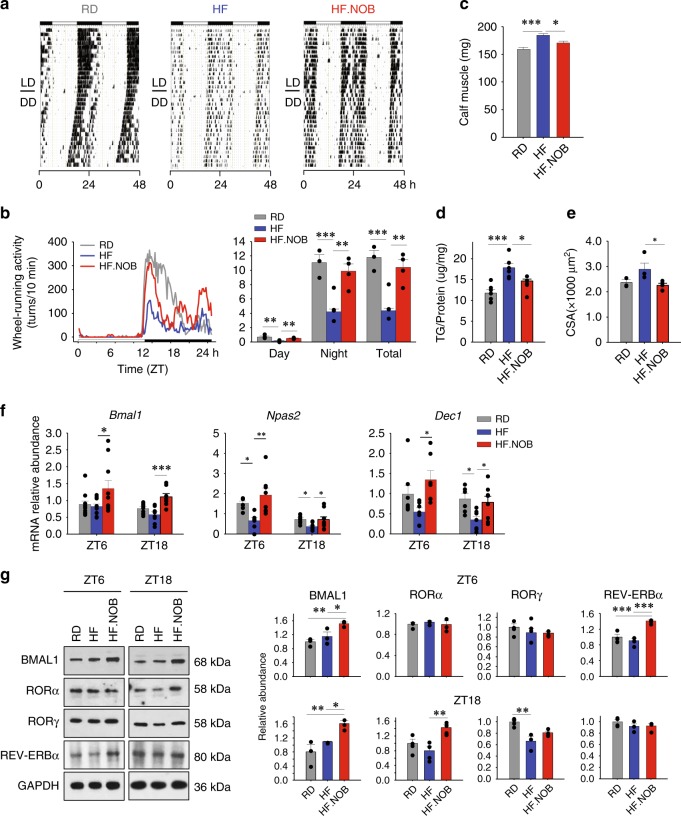
NOB enhanced circadian rhythms in skeletal muscle. Aged mice were fed with regular diet (RD), high-fat (HF) diet, or HF diet with NOB (HF.NOB). **a** Wheel-running activity (WRA) analysis. Representative actograms are shown (*n* = 4, 6, and 6 for RD, HF, and HF.NOB, respectively). **b** Circadian wheel-running activity was calculated from **a** by using the ActiView software (*n* = 4, 6, and 6 for RD, HF, and HF.NOB, respectively). **c** Calf muscle mass were measured after 20 weeks of treatment (*n* = 20, 15, and 15 for RD, HF, and HF.NOB, respectively). **d** Triglyceride contents in calf muscle (*n* = 7, 6, and 4 for RD, HF, and HF.NOB, respectively). **e** Fiber size (*n* = 3, 4, and 5 for RD, HF, and HF.NOB, respectively). **f** Expression of *Bmal1*, *Npas2*, and *Dec1* genes (ZT6, *n* = 11, 10, and 10 for RD, 10 HF, and 10 HF.NOB, respectively; ZT18, *n* = 9, 8, and 8 for RD, HF, and HF.NOB, respectively). **g** Representative BMAL1, RORα, RORγ, and REV-ERBα protein levels and quantification results are shown (*n* = 3). *p < 0.05, **p < 0.01, ***p < 0.001, one-way ANOVA. Data are presented as mean ± SEM in bar graphs

We next focused on the HFD groups to delineate circadian and physiological bases of NOB effects. In circadian wheel-running behavior assays, HFD-fed aged mice showed significant reduction in wheel activity and increase in free-running period length (Fig. 3a, b and Supplementary Fig. 3a)^24^ relative to RD-fed control mice. HF.NOB treatment markedly enhanced circadian wheel-running activity levels compared with HF and reversed the period lengthening by HF to a moderate yet statistically significant degree (Fig. 3a, b and Supplementary Fig. 3a). Interestingly, NOB accentuated activity peaks at both dawn and dusk, conferring a crepuscular circadian behavior (Fig. 3b).

Given our results here and previously reported^36^ showing NOB-mediated strong improvement in these muscle-related parameters, we reasoned that the skeletal muscle clock and function may be strengthened by NOB, which in turn contributes to overall fitness in aged mice. We found that calf muscle weight was elevated by HF and partially normalized by HF.NOB when compared with RD (Fig. 3c). We next measured lipid content in skeletal muscle. Both muscle TG measurements and Oil Red O staining showed exaggerated lipid accumulation in calf muscle from HFD-fed mice and reversal by HF.NOB (Fig. 3d and Supplementary Fig. 3b), suggesting muscle weight decrease by NOB was in part attributable to the reduction of fat accumulation. Consistently, muscle fiber size was normalized by NOB (Fig. 3e). As muscle lipid deposition can elicit deleterious consequences including lipotoxicity, blunted energy homeostatic activity, and insulin resistance, these results implicate skeletal muscle as a physiological target for NOB-mediated metabolic enhancement.

Our previous study showed that NOB activates RORs and enhances circadian clock amplitude^36^. To dissect the circadian mechanism underlying the heathy aging effects, we examined circadian gene expression in skeletal muscle (Fig. 3f and Supplementary Fig. 3c). Interestingly, several ROR target genes, including *Bmal1*, *Npas2*, and the clock output gene *Dec1*, were strongly upregulated in HF.NOB mouse skeletal muscle (Fig. 3f). Expression of *Bmal1* and *Dec1* was also among the clock genes found to be activated by NOB under the RD feeding condition (Supplementary Fig. 3d). At the protein level, we observed BMAL1 protein induction by NOB (i.e., compare HF and HF.NOB) at both ZT6 and ZT18 (Fig. 3g). RORα and RORγ proteins also showed varying degrees of induction at ZT18, whereas REV-ERBα protein was enriched at ZT6 by NOB treatment. Extending our previous results showing clock gene induction by NOB in the liver^36^, these data suggest a modulatory role of NOB-RORs in skeletal muscle circadian oscillator.

### NOB induces mitochondrial respiration and energy expenditure

**Fig. 4:**
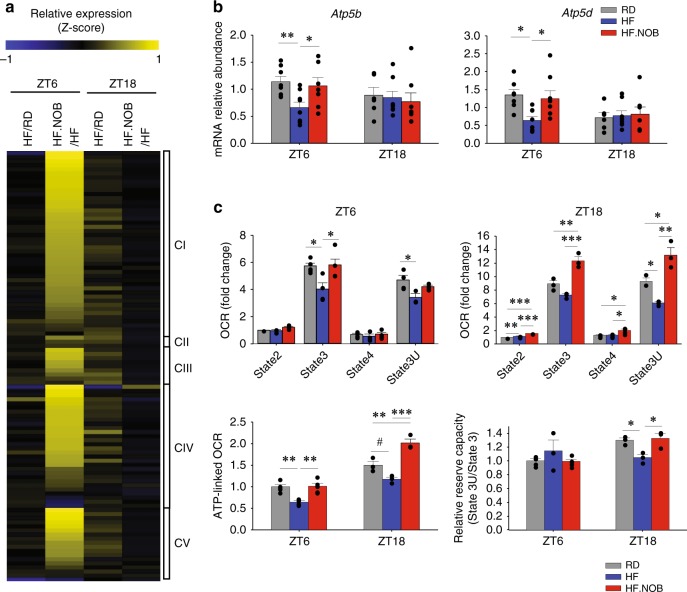
NOB activated mitochondrial OXPHOS gene expression and function. Aged mice were fed with regular diet (RD), high-fat (HF) diet, or HF diet with NOB (HF.NOB). **a** Heat map of pairwise expression comparison for mitochondrial respiration complex genes from RNA-sequencing analysis (*n* = 3). The *z*-score indicates the number of SDs away from the mean of expression. **b** Expression of two mitochondrial complex V genes known to be targets of RORs were restored by NOB (ZT6, *n* = 11, 10, and 10 for RD, HF, and HF.NOB, respectively; ZT18, *n* = 9, 8, and 8 for RD, HF, and HF.NOB, respectively). **c** Oxygen consumption rate (OCR), ATP-linked OCR, and relative reserve capacity in mitochondria isolated from calf muscle after 20 weeks of treatment. Average and representative assay results are shown (ZT6, *n* = 5, 4, and 5 for RD, HF, and HF.NOB, respectively; ZT18, *n* = 4). *p < 0.05, **p < 0.01, ***p < 0.001, one-way ANOVA; #*p* < 0.05, *t*-test. Data are presented as mean ± SEM in bar graphs

We next performed RNA-sequencing (RNA-seq) of skeletal muscle isolated from HF- and HF.NOB-treated aged mice, to delineate the transcriptomic changes. Pathway analysis revealed a strong effect of NOB on mitochondrial functions (Supplementary Fig. 4a). Expression of genes encoding components of MRCs I–V (CI–V) showed a moderate yet near-ubiquitous induction by NOB at ZT6 during the light/inactive phase (HF.NOB/HF; Fig. 4a), with 39% of genes showing >50% induction or more. In addition, a slight induction of a subset of MRC genes was observed in the HFD group at ZT18 relative to RD (Fig. 4a). These results suggest that gene upregulation occurs in the inactive, resting phase prior to food intake and peak mitochondrial respiration during the active phase.

Comparison of our RNA-seq results with previous chromatin immunoprecipittion sequencing studies^38,39^ identified several potential clock-regulated genes with E-box (CLOCK/BMAL1-binding site) and RORE (ROR and REV-ERB-binding site) promoter elements. In particular, the CV-related genes *Atp5b* and *Atp5d* contain RORE promoter elements bound by both RORα and RORy. In accordance, whereas HF reduced their expression, NOB enhanced it (Fig. 4b). On the other hand, several other genes with only CLOCK/BMAL1 or RORα binding showed no or slight upregulation in response to NOB (Supplementary Fig. 4b). Likewise, under the RD condition, effects of NOB on mitochondrial gene expression were generally modest or insignificant (Supplementary Fig. 4c).

We next performed extracellular flux analysis to investigate mitochondrial bioenergetics. Using pyruvate/malate as substrates, we found that skeletal muscle mitochondria isolated from HF.NOB-fed mice showed recovery of State 3 oxygen consumption rate (OCR) to RD levels, which was reduced by HF at ZT6, and at ZT18 the HF.NOB showed even greater OCR compared with RD (Fig. 4c). ATP-linked OCR, indicating ATP synthesis activity, was induced in HF.NOB relative to HF (Fig. 4c), suggesting NOB enhances ATP production by CV. In addition, reserved respiratory capacity, as measured by State 3U/State 3, was reduced by HF but restored by HF.NOB to RD levels at ZT18 (Fig. 4c). Using succinate/rotenone as substrates, we also observed upregulation of State 3U OCR (Supplementary Fig. 4d), indicating elevated Complex II (CII)-mediated respiration. In contrast, under RD feeding, skeletal muscle mitochondrial respiration was not significantly improved by NOB (Supplementary Fig. 4e). These results indicate a greater effect of NOB on mitochondrial respiration in HF than in RD, consistent with increased in vivo oxygen consumption in the HFD.NOB group (Fig. 2e).

### ROR-NOB augments muscle mitochondrial bioenergetics

**Fig. 5:**
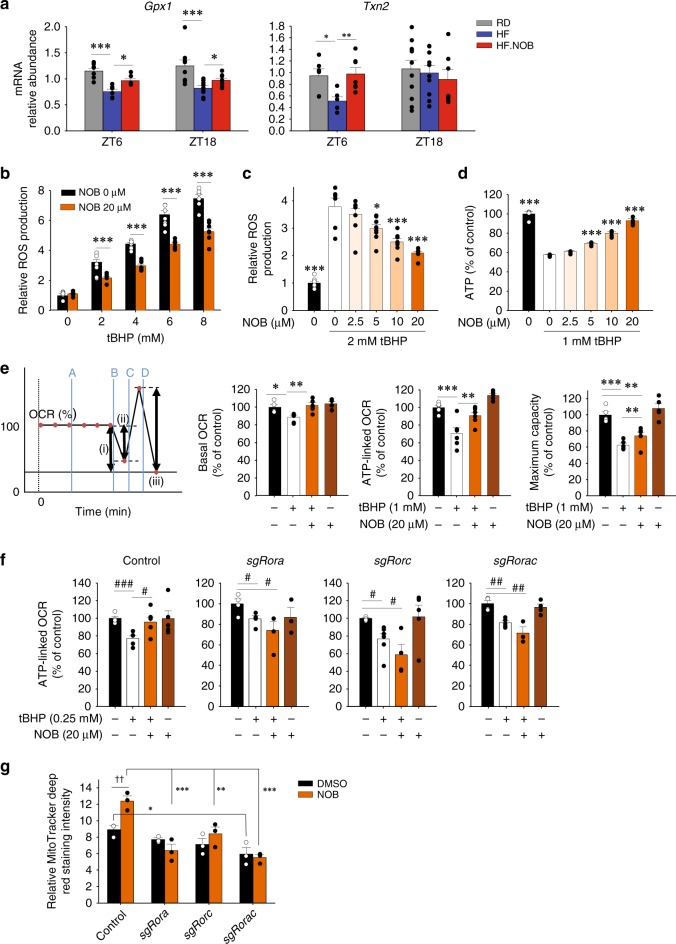
NOB improved mitochondrial OXPHOS function. **a** Real-time qPCR analysis revealed enhanced expression of RORE-containing anti-oxidant genes in skeletal muscle from aged mice treated with NOB (ZT6, *n* = 7, 6, and 6 for RD, HF, and HF.NOB, respectively; ZT18, *n* = 9, 9, and 7 for RD, HF, and HF.NOB, respectively). **b**–**g** Effects of NOB in C2C12 cells. **b**, **c** NOB-suppressed tert-butyl hydroperoxide (tBHP) induced ROS production (*n* = 6). **d** NOB rescued ATP production against tBHP-induced oxidative stress (*n* = 4). **e** NOB restored mitochondrial OXPHOS function (PortA; Vehicle or 1 mM tBHP, PortB; 2.5 μg/ml Oligomycin, PortC; 4 μM FCCP, PortD; 2 μM Antimycin A). Representative assay condition (line graph) and Basal OCR after tBHP treatment (left bar graph), ATP-linked OCR (middle bar graph), and maximum respiration capacity (right bar graph) (*n* = 5) are shown. **f** NOB effect was attenuated by RORs knockdown (*n* = 5). **g** MitoTracker deep red staining of control and ROR-deleted C2C12 cells (*n* = 3). Two-way ANOVA analysis shows statistically significant differences between treatments and genotypes. *p < 0.05, **p < 0.01, ***p < 0.001, one-way ANOVA; ^††^*p* < 0.01, two-way ANOVA; #*p* < 0.05, ##*p* < 0.01, ###*p* < 0.001, *t*-test. Data are presented as mean ± SEM in bar graphs

In addition to ATP production, mitochondrial respiration also produces ROS, which, if not controlled, causes cellular damage during aging. Therefore, we examined mRNA expression levels for anti-oxidative enzymes. With HF feeding, Glutathione peroxidase 1 (Gpx1) and Thioredoxin 2, both localized in the mitochondria and encoded by RORγ target genes, showed reduced levels in HF relative to RD and were enhanced by HF.NOB at ZT6 (Fig. 5a). Thioredoxin 1, mainly localized in the cytosol, and superoxide dismutases (Sod1, Sod2, and Sod3) did not show significant changes in mRNA expression levels (Supplementary Fig. 5a). In comparison, NOB showed overlapping effects on redox gene expression in RD, with significant changes in *Gpx1* and *Sod1* mRNA levels (Supplementary Fig. 5b).

To examine NOB’s effect against oxidative stress, we employed C2C12 myotubes, which harbor functional oscillators responsive to NOB (Supplementary Fig. 5c). We treated C2C12 myotubes with the oxidant tert-butyl hydroperoxide (tBHP). ROS production measured by H_2_DCFDA fluorometric assays in differentiated C2C12 myotubes was significantly suppressed by NOB treatment in a dose-dependent manner (Fig. 5b, c; tBHP and NOB dose responses, respectively). We next examined NOB effect on ATP synthesis under oxidative stress. ATP content in C2C12 cells treated with tBHP was significantly reduced (~50%), yet recovered by NOB treatment in a dose-dependent manner (Fig. 5d). These results indicated that NOB guards against oxidative stress, while maintaining robust ATP production. In accordance, extracellular flux analysis showed that oxidative stress provoked by tBHP impaired basal OCR, ATP-linked OCR, and maximum capacity of mitochondrial respiration, which was reversed by NOB (Fig. 5e).

We next performed CRISPR to generate ROR-deficient C2C12 cells. As shown in Fig. 5f, ATP-linked OCR was enhanced by NOB in wild-type control C2C12 cells, whereas cells deficient in *Rora*, *Rorc*, or *Rora/c* following CRISPR-guided DNA disruption failed to respond to NOB, to induce OCR (compare lanes 2 and 3 for each graph). We next carried out MitoTracker staining to evaluate mitochondrial content. Similar to OCR measurements above, mitochondrial volume was increased in NOB-treated control C2C12 cells, whereas ROR deficiency, individually or combined, abrogated NOB-dependent increase (Fig. 5g and Supplementary Fig. 5d). Together, these functional assays using CRISPR-generated ROR-deficient C2C12 cells established ROR requirement for the observed mitochondrial enhancement by NOB.

### Metabolomic landscape in aged skeletal muscle

**Fig. 6:**
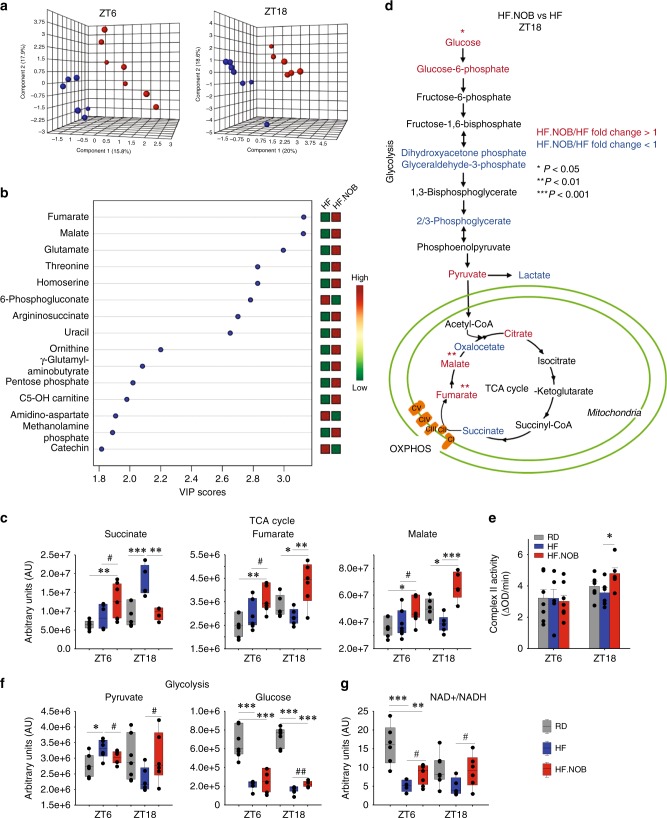
NOB regulates TCA and glycolysis flux. Aged skeletal muscle was subjected to unbiased metabolomic analysis. **a**, **b** PLS-DA analysis results are 3D plotted for ZT6 and ZT18, and VIP score plotting for overall are shown. Blue and red dots indicate individual HF and HF.NOB-fed mice (ZT6, *n* = 7 and 8 for HF and HF.NOB, respectively; ZT18, *n* = 7 and 6 for HF and HF.NOB, respectively). **c** TCA cycle metabolites in skeletal muscle (ZT6, *n* = 7, 7, and 8 for RD, HF, and HF.NOB, respectively; ZT18, *n* = 7, 7, and 6 for RD, HF, and HF.NOB, respectively). **d** Glycolysis and TCA pathway analysis between HF and HF.NOB in skeletal muscle at ZT18. Red and blue indicate fold change ≥ 1.1 and ≤ 0.9, respectively (*n* = 7 and 6 for HF and HF.NOB, respectively). **e** Complex II activity in isolated mitochondria from calf muscle were measured (ZT6, *n* = 7, 6, and 8 for RD, HF, and HF.NOB, respectively; ZT18, *n* = 6). **f** Glycolysis-related metabolites in skeletal muscle (ZT6, *n* = 7, 7, and 8 for RD, HF, and HF.NOB; ZT18, *n* = 7, 7, and 6 for RD, HF, and HF.NOB, respectively). **g** NAD+ /NADH in skeletal muscle (ZT6, *n* = 7, 7, and 8 for RD, HF, and HF.NOB, respectively; ZT18, *n* = 7, 7, and 6 for RD, HF, and HF.NOB, respectively). *p < 0.05, **p < 0.01, ***p < 0.001, one-way ANOVA; #*p* < 0.05, ##*p* < 0.01, *t*-test. Data are presented as mean ± SEM in bar graphs. For box-whisker plots, box edges correspond to 25th and 75th percentiles, lines inside the box correspond to 50th percentiles, and whiskers include extreme data points

To interrogate metabolic homeostasis, we conducted unbiased metabolomics using skeletal muscle tissues collected at ZT6 and ZT18. RD samples showed clear segregation from HF and HF.NOB, indicating a strong dietary effect (Supplementary Fig. 6a). However, pairwise comparison between HF and HF.NOB also revealed strong clustering between the two sample groups at both circadian time points (Fig. 6a and Supplementary Fig. 6a), affirming an important modulatory role of NOB in aged muscle metabolism. Consistent with functional results above, we observed prevalent changes in metabolites involved in mitochondrial metabolism. Partial least-squares Discriminant Analysis (PLS-DA) and corresponding Variable Importance in Projection (VIP) analysis, which evaluates and ranks the contribution of metabolites to PLS-DA variance based on the weighted sum of squares for PLS loadings, revealed most robust changes in several tricarboxylic acid (TCA) cycle metabolites (Fig. 6b). Specifically, although several other TCA intermediates showed no clear NOB-dependent changes at either circadian time points (Supplementary Fig. 6b), fumarate and malate showed a trend of decrease at ZT18 in HF relative to RD, and were strongly enriched in HF.NOB (Fig. 6c). In contrast, the preceding TCA metabolite succinate exhibited a striking accumulation at ZT18 in HF and reverted to RD levels by NOB (Fig. 6c). This contrasting pattern suggested a focal regulation on succinate dehydrogenase or CII of the ETC (Fig. 6d and Supplementary Fig. 6c). CII represents the sole point of direct interaction between TCA and ETC. We conducted enzyme-linked immunosorbent assays (ELISAs) to measure CII activity in skeletal muscle mitochondria from aged mice (Fig. 6e). Although HF treatment showed a trend of reducing CII activity relative to RD, HF.NOB significantly elevated the activity levels compared with both RD and HF. This result illustrates a direct effect of NOB on the activity of an MRC complex, consistent with Seahorse analysis showing increased CII-dependent respiration.

Levels of pyruvate, the key metabolite connecting glycolysis in the cytosol and TCA, were decreased and increased by NOB at ZT18 and ZT6, respectively (Fig. 6f). In comparison, intracellular glucose levels were only moderately changed by NOB treatment (Fig. 6f), suggesting dynamic changes in other glycolysis intermediates (Supplementary Fig. 6d). NADH is a substrate for mitochondrial respiration and NAD+ is a substrate for glycolysis and an energy indicator important for aging. Whereas the levels of NAD+ and NADH did not show major changes, the NAD+/NADH ratio was reduced in HF at both ZT6 and ZT18, and significantly enhanced by NOB treatment (Fig. 6g), consistent with elevated respiratory activities in HF.NOB than in HF.

### NOB modulates MRC architecture

Recent studies have provided interesting insights into the architectural reorganization of MRCs, specifically formation of SCs in mammalian mitochondria mainly composed of Complex I, (NADH:ubiquinone oxidoreductase), Complexes III (III2, cytochrome bc1 complex; ubiquinol cytochrome c oxidoreductase), and Complex IV (cytochrome c oxidase)^40^. Previously, SC formation was found to increase with aging in rat skeletal muscle^6^, perhaps functioning as a compensatory mechanism to maintain respiratory function during aging.

**Fig. 7:**
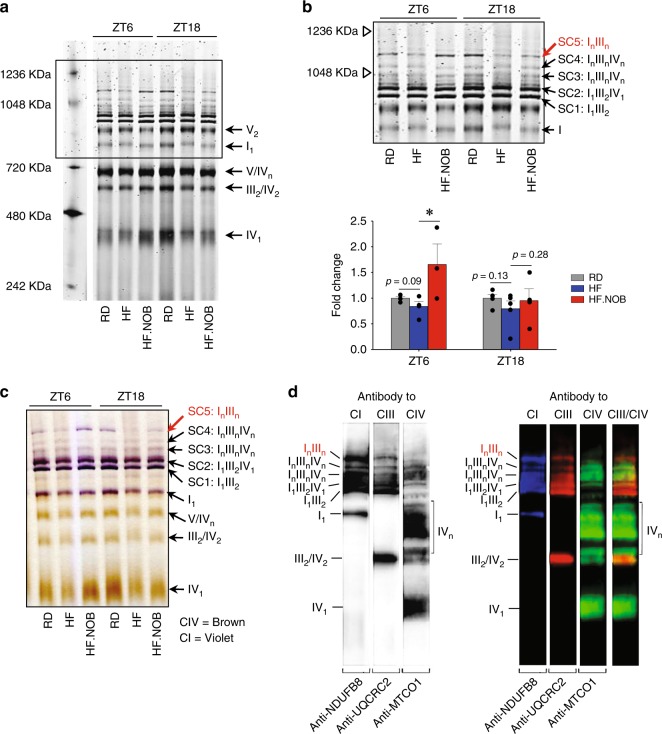
NOB restored mitochondrial supercomplex formation affected by HF feeding. Aged mice were fed with regular diet (RD), high-fat (HF) diet, or HF diet with NOB (HF.NOB). **a** Mitochondrial OXPHOS supercomplexes (SCs) analyzed by BN-PAGE. Representative BN-PAGE of digitonin-solubilized mitochondria purified from skeletal muscle of RD, HF, and HF-NOB-treated mice is shown. **b** Upper panel: enlarged view of the box area from **a**. Lower panel: quantification of SC5 band intensities (ZT6, *n* = 4, 4, and 3 for RD, HF, and HF.NOB, respectively; ZT18, *n* = 5, 5, and 4 for RD, HF, and HF.NOB, respectively). **c** In-gel activity assay with Complex IV and subsequently with Complex I substrates performed as described in Methods. Brown and violet color bands indicate CIV and CI activity, respectively. **d** Western blot analysis of the respiratory Complexes I, III and IV isolated from skeletal muscle mitochondria in aged mice. Western blotting was performed as described in Methods. Lane 1: antibody to CI subunit 8, pseudo-colored blue; lane 2: antibody to CIII, subunit 2 (QCR2, Cor2), pseudo-colored red; lane 3: antibody to CIV subunit I (Cox1), pseudo-colored green; lane 4: overlay of lanes 2 and 3. Of note, in SC labels, *n* reflects the number of individual complexes in SCs, not the level of their oligomerization. *p < 0.05, one-way ANOVA. Data are presented as mean ± SEM in bar graphs

We therefore examined MRC architecture and SC formation in aged skeletal muscles under the HF condition where we observed robust NOB effects on mitochondrial respiration. After digitonin extraction, the MRCs and SCs were separated by blue native polyacrylamide gel electrophoresis (BN-PAGE) (Fig. 7a, b) and analyzed by in-gel enzyme activity staining (Fig. 7c) or western blotting (Fig. 7d) with antibodies against subunits of CI, CIII, and CIV. SC1 and SC2 (I_1_III_2_ and I_1_III_2_IV_1_, respectively) (Fig. 7b–d) were identified in the gel in good agreement with previously published data^41–43^. SC3 and SC4 contained CI, CIII, and CIV, and are labeled as I_n_III_n_IV_n_. Interestingly, the largest SC5 were found to be devoid of CIV (therefore denoted as I_n_III_n_) and displayed a similar migration pattern with a previously reported SC containing two CI^44,45^. Whereas HF feeding reduced levels of the SC5 relative to RD and the HF.NOB group showed the highest abundance at ZT6 (Fig. 7b, c). These results suggest a large SC species, perhaps with an enriched CI stoichiometry, is regulated by NOB in a time-dependent manner.

## Discussion

In the current study, we show that NOB, an ROR agonist compound, strengthens metabolic fitness and physical activity in normally aged mice. Furthermore, when aged mice were challenged with HF diet, akin to retirees exposed to nutritional excess, NOB exerted profound efficacies to promote healthy aging as evidenced by improved energy expenditure, cold tolerance, exercise endurance, grip strength, and inflammatory markers. Multiple lines of evidence, including RNA-seq, extracellular flux analysis and cellular functional assays, metabolomic profiling, and biochemical characterization of ETC complexes, underscore a concerted optimization of MRC function and architecture in aged skeletal muscle by NOB in the HF feeding condition. In comparison, NOB was effective on a smaller, overlapping set of physiologic parameters in RD-fed aged mice. Of note, in our previous study^36^ NOB showed essentially no beneficial effects in RD-fed young mice, suggesting the current NOB effects in the RD condition are aging-related. Consistent with a role of RORs and the circadian clock, beneficial effects were dependent on RORs and/or circadian timing. Together, our study demonstrates that pharmacological circadian manipulation can coordinately promote healthy aging.

Aging-related energy imbalance is accompanied by attenuation of circadian rhythms at physiological and behavioral levels. Our study highlights the importance of robust energy expenditure during aging. NOB was previously reported to activate RORs, to enhance circadian oscillation and protect against metabolic disorders^36,37,46^. Notably, NOB was able to elevate energy expenditure in both diet-induced obese mice and *db/db* diabetic mice^36^, an efficacy that requires a functional circadian clock. Here we observed NOB-dependent increase in energy expenditure in aged mice, particularly under HF conditions. As a result, a broad array of heathy aging markers were strongly improved in aged mice fed with HF.NOB. These findings suggest that a key output function of circadian potentiation may entail the enhancement of energy expenditure, a notion consistent with previous time-restricted dietary intervention studies^13,25^.

This study highlights skeletal muscle mitochondria as a pivotal target for circadian enhancement of energy expenditure. Skeletal muscle is the major site of glucose and lipid uptake where the clock has been shown to regulate glucose metabolism, mitochondrial function, and muscle physiology^10,11,47,48^. Here we showed strong effects of NOB on ROR-targeted circadian gene expression and revealed improved core body temperature, energy expenditure, as well as exercise endurance. These effects are consistent with an important role of RORs in metabolic regulation in skeletal muscle^47,49^. RNA-seq analysis of skeletal muscle transcription unveiled a prevalent induction of MRC gene expression at ZT6 by NOB, likely in preparation for the beginning of dark phase when a surge of food intake and metabolic activities will commence. Our analyses revealed comprehensive enhancement of mitochondrial function in skeletal muscle, including enhanced mitochondrial content and ATP production coupled with decreased ROS production. Biochemical and metabolomic studies further revealed optimized activity of MRCs. Both elevated CII (succinate dehydrogenase) activity and enrichment of SC5 (I_n_III_n_) could conceivably transfer electrons to CIII more efficiently. Although not a full respirasome, SC5 is expected to functionally cooperate with free CIV for efficient electron transfer^40^. Association of CI and CIII is also expected to stabilize CI and diminish oxidative stress, particularly with CI and CIII as the main sites of ROS production^50,51^. Of note, whereas most characterized SCs contain only one copy of CI, SC5 showed similar migratory behaviors with large SCs with I_2_ stoichiometry^44^. Our finding of this partial respirasome in skeletal muscle that is regulated by aging, diet, and a clock-modulating compound may provide a physiological context to determine the functional significance of SCs containing multiple CI (I_n_) in future studies. Finally, although it was previously demonstrated that the C57BL/6 mouse strain expresses a truncated isoform of the assembly protein SCAF1/COX7A2L^52^, our results are consistent with the reported normal assembly of the SCs composed of CI, CIII, and CIV, or CI and CIII in the heart and skeletal muscle of these mice^3,42,43^. Although the underlying mechanisms will require further study, our results together indicate a focal activation of mitochondrial respiration by NOB to decelerate metabolic aging.

NOB was previously shown to activate RORs and enhance circadian amplitude in vitro (PER2::LUC reporter) and in vivo (wheel-running behavior and clock gene expression in HF liver)^36^. The current study showed upregulation of ROR target genes involved in circadian rhythm, mitochondrial ETC and ROS scavenging, especially under the HF feeding condition. Further, several aspects of mitochondrial enhancement by NOB were found to require RORs in CRISPR experiments. In conjunction with our previous detailed molecular characterization^36^, these results support a pivotal role of RORs to mediate the circadian effects of NOB. Ongoing studies in our lab using conditional *Ror* knockout mice will provide further in vivo functional insight into the NOB-ROR axis. The exact regulatory pathways emanating from and governed by the ROR-NOB axis in aging also remain to be elucidated. Specifically, how does activation in the core oscillator via RORs propagate to physiological improvement and healthy aging? Earlier studies reported dysregulation of clock gene expression in peripheral metabolic tissues^15,17,53^, whereas strong circadian gene oscillation appeared to be maintained in central clock neurons^17,53^. More recent transcriptomic studies provide evidence that the core circadian gene expression was not significantly affected in peripheral tissues^14^, suggesting signal transduction or amplification renders clock-controlled downstream pathways dysfunctional during aging. A number of metabolic pathways are known to be regulated by circadian clocks and play key roles in aging. For example, CR involves several nutrient-sensing pathways including AMPK, AKT, and mTORC1, all of which have been reported to functionally interact with the clock^29,54^. Our metabolomic studies showed a NOB-dependent increase in the NAD+/NADH ratio in aged skeletal muscle. The NAD+-dependent deacetylase Sirtuin (SIRT) proteins play important roles at the interface of energy homeostasis, clock and aging^30^. Mammals express seven SIRT proteins (SIRT1–7), several of which have been implicated in circadian regulation of metabolism^55,56^.

The robust healthy aging effects described herein provide a strong rationale for a more rigorous test of lifespan effects in future studies. Modeling after the gold standard established by the National Institute on Aging (NIA) Intervention Testing Program^57–59^, future survival studies should take into account various factors including sex, genetic background, starting age, a predetermined sample size, diet composition, and dose. In the current study, we reported a pilot survival experiment using aged male mice fed with normal diets with or without NOB, starting at 16 months of age. The results showed a possible median lifespan extension with no effects on the maximum lifespan. This result, although somewhat surprising, is reminiscent of several recent dietary intervention studies where strong health benefits do not translate into maximum lifespan extension^60–62^. The reason for this uncoupling is unclear, but it has been postulated that dietary treatment may have age- or treatment duration-dependent effects^61^. For example, 4-day fasting-mimicking diet cycles were found to be beneficial in mid-age and old mice (16–26 months), but not in very old (>26.5 months) mice^60^. Likewise, in human studies, protein restriction has been shown to be protective against mortality in 65 years and younger, but not 66 years and older, subjects^63^. A comprehensive survival study in the future will be highly informative to address this emerging issue.

In conclusion, we demonstrate a potent efficacy of an ROR agonist, NOB, to promote circadian metabolism and healthy aging against metabolic challenge in mice, and functional and mechanistic studies revealed MRC activity and architecture in skeletal muscle as a nodal target of the NOB-ROR axis. Our study thus unveils a promising pharmacological strategy at the interface of circadian clock, metabolic fitness, and healthy aging.

## Methods

### Animal studies

For animal studies, except the longevity experiment, 20- to 22-month-old male C57BL/6 mice from aged mouse colony at the NIA and 10-week-old male C57BL/6 mice from the Jackson Laboratory (#000664) were used as aged and young mice, respectively. After 1 week of acclimation, animals were divided to different diet groups. In the HFD feeding experiments, HF and HF.NOB were purchased from Research Diets (D12492 and D14081401, 0.1% NOB). The control regular-fat diet was Purina 5053 (Pico Lab). In the RD feeding experiments, regular-fat diets equivalent with Purina 5053 in macronutrients were purchased from Research Diets for both RD and RD.NOB (D08112304 and D14081401, 0.1% NOB). Phenotyping comparison showed no significant difference in physiological parameters tested in aged mice fed with the two RD diets (Purina 5053 and D08112304). NOB was obtained from commercial sources (GenDEPOT and Selleck Chem.). Body weight was monitored weekly by 12 weeks of treatment. All other metabolic, physiological, and behavioral assays were performed after a minimum of 8 weeks of treatment. Caloric intake was measured at ZT0 and ZT12 for 1 week and daily caloric intake was derived. Core body temperature was measured by using a rectum probe (ThermoWorks). For cold tolerance test, the initial body temperature was measured before the mice were transferred to 4 °C. Core body temperature was measured every hour by using a rectum probe (ThermoWorks). End-point body weight and tissue weights were measured after 20–22 weeks of treatment. Calf muscle used throughout this study refers to mixed type gastrocnemius and soleus muscles. All animal studies were approved by UTHealth Center for Laboratory Animal Medicine and Care (CLAMC) and were conducted in compliance with CLAMC-designated guidelines. The mouse facility is specific-pathogen free and is tested with sentinel mice monthly.

### C2C12 cell culture, differentiation, and CRISPR

C2C12 myoblast cells (ATCC, CRL-1772) were maintained in Dulbecco’s modified Eagle medium (DMEM) with 10% fetal bovine serum and penicillin/streptomycin until 80–90% confluence. For differentiation, cells were incubated in differentiation media (DM; DMEM containing 2% horse serum and penicillin/streptomycin). DM was changed daily until cells were fully differentiated (around day 5). To generate Rora, Rorc, and Rorac CRISPR cell lines, the sense and antisense guide RNAs were designed using the https://crispr.dbcls.jp/ program (Supplementary Table 1) and cloned into the BsmB1 site of the GeCKO vector^64^. To examine clock gene expression, C2C12 cells were differentiated in DM until day 3. Cells were treated with 100 nM of Dexamethasone for 1.5 h for synchronization. After release (CT0), cells were treated with dimethyl sulfoxide or 20 µM NOB, and collected and lysed in TRIzol reagent every 4 h. Samples were stored in −80 °C before quantitative PCR (qPCR) analysis.

### Glucose tolerance test

Glucose tolerance test was conducted as follows^36,37^. After overnight fasting, animals were weighed prior to glucose injection (1 mg/kg body weight). Blood glucose was measured at 0, 15, 30, 60, and 120 min after glucose challenge.

### Serum content assays

Mouse serum samples were collected at the indicated circadian times (ZT6 and ZT18). Serum-free glycerol and TG were measured by colorimetric assays (Sigma). Serum FFAs were measured by colorimetric assays (BioVision). ELISA tests were performed to measure serum inflammation markers including LBP (LSBio), IL-6, and TNFα (Thermo Fisher).

### Circadian activity and period measurement

Circadian activity and periods were recorded by VitalView circadian chamber system and analyzed by the ActiView software (Starr Life Sciences). Wheel-running activity was quantified as an average of 7-day data collection.

### Muscle fatigue test

Muscular endurance test was performed by using a motorized treadmill (Treadmill Simplex II, Columbus Instruments) with 5% incline. Mice were acclimated for 4 days followed by 7 days of recovery. After recovery, mice were placed on the treadmill with the speed of 6 m/min. The running speed were increased every 5 min with 1 m/min until the mice showed fatigue defined by an inability to return to the treadmill or staying on the electrical shock grids for 10 s.

### Grip strength test

Grip strength test was performed by using a BIO-GS3 Grip Test instrument (Bioseb). Each animal was subjected to five trials each for forelimb and hindlimb, and average values were calculated.

### Noninvasive piezoelectric transducer sleep/wake recording

Sleep/wake recording was done by using a noninvasive piezoelectric transducer sleep/wake recording system (Signal Solutions, Inc.). Briefly, animals were single-housed in the Piezo system with free access for food and water under 12:12 light:dark cycles. The initial 48 h acclimation period was followed by actual data recording for 3 days.

### RNA-sequencing

Total RNA was extracted from frozen calf muscle by applying TRizol method (Invitrogen). Two micrograms of extracted RNA was used for Illumina RNA-seq analysis. RNA-seq data were aligned to mouse reference genome (mm10) using Tophat2 v2.0.12^65^. The abundance of genes was quantified by Cufflinks v2.2.1^66^.

### Real-time qPCR

Total RNA was extracted from frozen calf muscle by applying TRizol method (Invitrogen). Two micrograms of extracted RNA were used for cDNA synthesis. Gene expression was analyzed by using Mx3000p (Agilent technologies). Primer sequences are listed in the Supplementary Information (Supplementary Table 2).

### Histological and cytological staining

Isolated calf muscles were fixed in 10% formalin overnight at 4 °C followed by replacement with 70% EtOH and maintaining at 4 °C minimum overnight. Tissues were processed with paraffin embedding, sectioning, and placed on slides. Dewaxed sections were processed with regular hematoxylin and eosin staining. For Oil red O staining, isolated calf muscle was snap frozen in liquid nitrogen and then stored in −80 °C. Tissues were embedded in OTC compound and sectioned with Cryostat in 12 μm thickness and placed on slides. Sections on slides were rounded with liquid blocker pen. Sections were covered with Oil red O solution and incubated at room temperature for 10 min. Then slides were rinsed with running tap water for 30 min. Sections were captured as bright-field images with a light microscope. MitoTracker deep red staining (Thermo Fisher) was performed based on the manufacturer’s protocol.

### Metabolomic analysis

Isolated calf muscle tissue was flash frozen in liquid nitrogen and stored at −80 °C until analysis. Prior to liquid chomatography analysis, samples were placed on ice and suspended with methanol:acetonitrile:water (5:3:2, v-v) to a concentration of 30 mg/ml. Glass beads (GB10, Next Advance, Troy, NY, USA) were added to each tube and placed into a Bullet Blender (Next Advance, Troy, NY, USA) at setting 3 for 5 min at 4 °C to homogenize tissue. Suspensions were then vortexed continuously for 30 min at 4 °C. Insoluble material was removed by centrifugation at 10,000 × *g* for 10 min at 4 °C and supernatants were isolated for metabolomics analysis by UHPLC-MS analysis^67^. Acquired data were then converted from.raw to.mzXML file format using Mass Matrix (Cleveland, OH, USA). Samples were analyzed in randomized order with a technical mixture injected after every 15 samples to qualify instrument performance. Metabolite assignments, isotopologue distributions, and correction for expected natural abundances of deuterium, ^13^C, and ^15^N isotopes were performed using MAVEN (Princeton, NJ, USA)^68^. Graphs, heat maps, and statistical analyses (either *T*-test or analysis of variance (ANOVA)), metabolic pathway analysis, PLS-DA, and hierarchical clustering was performed using the MetaboAnalyst 3.0 package (www.metaboanalyst.com). Hierarchical clustering analysis was also performed through the software GENE-E (Broad Institute, Cambridge, MA, USA). XY graphs were plotted through GraphPad Prism 5.0 (GraphPad Software, Inc., La Jolla, CA, USA). See Supplementary Methods for additional details.

### Mitochondria isolation and biochemical analyses

Mitochondrial fractions were isolated from fresh calf muscle by the Potter–Elvehjem method. Purified mitochondria were solubilized in digitonin according to the procedure^69^ with minor modifications. To extract the respiratory SCs, mitochondria purified from the muscle tissue were solubilized by mild detergent digitonin (8 g/g of protein)^7^. After digitonin extraction, the respiratory chain complexes and SCs were separated by BN-PAGE and subjected to in-gel enzyme activity staining or western blot analysis with antibodies against subunits of Complexes I, III, and IV. Positions of Complex V (F0F1 ATPase) and its dimer (V2) were also identified by in-gel enzyme activity staining^41^. Mitochondrial lysates were subjected to BN-PAGE in a 3–12% acrylamide gradient gel in Bis-Tris buffer (Invitrogen) according to manufacturer’s instructions. After electrophoresis, gels were either stained with Imperial stain (Thermo Fisher Scientific) and Bio-Safe Coomassie G-250 (Bio-Rad) or used for western blotting analysis. For CI and CIV in-gel activity staining, mitochondrial lysates were subjected to BN-PAGE^41^. See Supplementary Methods for additional details.

### Seahorse mitochondrial OCR analysis

Isolated mitochondria were applied to Seahorse Bioanalyzer to determine bioenergetic flux. For Rora and Rorc individual and double knockdown (RoraKD, RorcKD, and RoracKD) C2C12 cells, cells were seeded on a V7 assay plate and maintained for 24 h. Cells were then pre-treated with 20 µM NOB in culture media for 24 h. See Supplementary Information for additional details.

### Western blotting

Western blotting for circadian proteins was performed using established methods^37^. Western blotting for the detection of the respiratory complexes CI, CIII, and CIV after BN-PAGE was performed using established methods^69^. See Supplementary Methods for details and Supplementary Fig. 7 for uncropped images.

### ROS detection

Differentiated C2C12 cells (DM5) were pre-treated with or without NOB and incubated for 24 h followed by various concentration of tBHP treatment in serum-free media for 2 h. After treatment, cells were treated with 20 μM H_2_DCFDA with 0.5 µg/ml Hoechst 33342 and further incubated for 30 min. Cells were rinsed and further incubated for 10 min for cells recovery. Finally, cells were rinsed with ice-cold phosphate-buffered saline (PBS) once and fluorescence were measured in PBS [DCF; ex: 485/em: 530 nm, Hoechst 33342; ex: 350/em:461].

### ATP contents measurement

We measured ATP content in tissue or culture cells by using an established protocol as follows^70^. In brief, C2C12 cells (DM5) were pre-treated with or without NOB and incubated for 24 h followed by various concentration of tBHP treatment in serum-free media for 1 h. C2C12 cells were lysed in 1.0 mL of ice-cold phenol-TE after treatment. Two hundred microliters of chloroform and 150 µL of de-ionized water were added. The homogenate was thoroughly shaken for 20 s and centrifuged at 10,000 × *g* for 5 min at 4 °C. Transfer supernatant onto new tubes on ice. ATP contents were measured with ATP determination kit (Molecular Probes, Inc., Eugene,OR).

### Mouse longevity study

Male mice (16-month-old) were obtained from the NIA rodent colony. Following 1 week of acclimation, mouse diets were replaced with the indicated diets, including RD and RD with 0.1% NOB supplement (RD.NOB, Research Diets) and monitor until death without experimental manipulation. Data were analyzed by the Kaplan–Meier method.

### Quantification and statistical analysis

Results are presented as mean ± SEM unless otherwise stated. All *n* numbers refer to biologically independent samples. Data were analyzed using Student’s *t*-test, one-way ANOVA followed by post-hoc analysis using Dunnett’s multiple comparison test or two-way ANOVA followed by post-hoc analysis using Bonferroni test as appropriate. For survival curve, we conducted Log-rank test and Mann–Whitney *U*-test. A value of *p* < 0.05 was considered statistically significant.

### Reporting summary

Further information on research design is available in the Nature Research Reporting Summary linked to this article.

## Supplementary information

Supplementary Information

Reporting Summary

Source Data

## Data Availability

All data that support the findings of the current study are available from the corresponding author upon reasonable request. The GEO accession ID for the RNA-seq dataset is GSE134304 [https://www.ncbi.nlm.nih.gov/geo/query/acc.cgi?acc = GSE134304]. The uncropped images of key western blottings are presented in Supplementary Fig. 7. The source data underlying the graphs are provided in the Source Data Excel file.

## References

[1] Lopez-Otin C, Blasco MA, Partridge L, Serrano M, Kroemer G (2013). The hallmarks of aging. Cell.

[2] Pannemans DL, Westerterp KR (1995). Energy expenditure, physical activity and basal metabolic rate of elderly subjects. Br. J. Nutr..

[3] Milenkovic D, Blaza JN, Larsson NG, Hirst J (2017). The enigma of the respiratory chain supercomplex. Cell Metab..

[4] Schagger H, Pfeiffer K (2000). Supercomplexes in the respiratory chains of yeast and mammalian mitochondria. EMBO J..

[5] Genova ML, Lenaz G (2015). The interplay between respiratory supercomplexes and ROS in aging. Antioxid. redox Signal..

[6] Lombardi A (2009). Defining the transcriptomic and proteomic profiles of rat ageing skeletal muscle by the use of a cDNA array, 2D- and Blue native-PAGE approach. J. Proteomics.

[7] Frenzel M, Rommelspacher H, Sugawa MD, Dencher NA (2010). Ageing alters the supramolecular architecture of OxPhos complexes in rat brain cortex. Exp. Gerontol..

[8] Takahashi JS (2017). Transcriptional architecture of the mammalian circadian clock. Nat. Rev. Genet..

[9] Kalsbeek A, la Fleur S, Fliers E (2014). Circadian control of glucose metabolism. Mol. Metab..

[10] Harfmann BD, Schroder EA, Esser KA (2015). Circadian rhythms, the molecular clock, and skeletal muscle. J. Biol. Rhythms.

[11] Andrews JL (2010). CLOCK and BMAL1 regulate MyoD and are necessary for maintenance of skeletal muscle phenotype and function. Proc. Natl Acad. Sci. USA.

[12] Brown SA, Pagani L, Cajochen C, Eckert A (2011). Systemic and cellular reflections on ageing and the circadian oscillator: a mini-review. Gerontology.

[13] Manoogian ENC, Panda S (2017). Circadian rhythms, time-restricted feeding, and healthy aging. Ageing Res Rev..

[14] Sato S (2017). Circadian reprogramming in the liver identifies metabolic pathways of aging. Cell.

[15] Banks G, Nolan PM, Peirson SN (2016). Reciprocal interactions between circadian clocks and aging. Mamm. Genome.

[16] Yamazaki S (2002). Effects of aging on central and peripheral mammalian clocks. Proc. Natl Acad. Sci. USA.

[17] Luo W (2012). Old flies have a robust central oscillator but weaker behavioral rhythms that can be improved by genetic and environmental manipulations. Aging Cell.

[18] Davidson AJ (2006). Chronic jet-lag increases mortality in aged mice. Curr. Biol..

[19] Hurd MW, Ralph MR (1998). The significance of circadian organization for longevity in the golden hamster. J. Biol. Rhythms.

[20] Kondratov RV, Kondratova AA, Gorbacheva VY, Vykhovanets OV, Antoch MP (2006). Early aging and age-related pathologies in mice deficient in BMAL1, the core componentof the circadian clock. Genes Dev..

[21] Gutman R, Genzer Y, Chapnik N, Miskin R, Froy O (2011). Long-lived mice exhibit 24 h locomotor circadian rhythms at young and old age. Exp. Gerontol..

[22] Patel SA, Chaudhari A, Gupta R, Velingkaar N, Kondratov RV (2016). Circadian clocks govern calorie restriction-mediated life span extension through BMAL1- and IGF-1-dependent mechanisms. FASEB J..

[23] Gill S, Le HD, Melkani GC, Panda S (2015). Time-restricted feeding attenuates age-related cardiac decline in *Drosophila*. Science.

[24] Kohsaka A (2007). High-fat diet disrupts behavioral and molecular circadian rhythms in mice. Cell Metab..

[25] Hatori M (2012). Time-restricted feeding without reducing caloric intake prevents metabolic diseases in mice fed a high-fat diet. Cell Metab..

[26] Katewa SD (2016). Peripheral circadian clocks mediate dietary restriction-dependent changes in lifespan and fat metabolism in *Drosophila*. Cell Metab..

[27] Acosta-Rodriguez VA, de Groot MHM, Rijo-Ferreira F, Green CB, Takahashi JS (2017). Mice under caloric restriction self-impose a temporal restriction of food intake as revealed by an automated feeder system. Cell Metab..

[28] Patel SA, Velingkaar N, Makwana K, Chaudhari A, Kondratov R (2016). Calorie restriction regulates circadian clock gene expression through BMAL1 dependent and independent mechanisms. Sci. Rep..

[29] Longo VD, Panda S (2016). Fasting, circadian rhythms, and time-restricted feeding in healthy lifespan. Cell Metab..

[30] Tevy MF, Giebultowicz J, Pincus Z, Mazzoccoli G, Vinciguerra M (2013). Aging signaling pathways and circadian clock-dependent metabolic derangements. Trends Endocrinol. Metab..

[31] Chen Z, Yoo SH, Takahashi JS (2018). Development and therapeutic potential of small-molecule modulators of circadian systems. Annu Rev. Pharm. Toxicol..

[32] Kojetin DJ, Burris TP (2014). REV-ERB and ROR nuclear receptors as drug targets. Nat. Rev. Drug Discov..

[33] Hirota T, Kay SA (2009). High-throughput screening and chemical biology: new approaches for understanding circadian clock mechanisms. Chem. Biol..

[34] Chen Z (2012). Identification of diverse modulators of central and peripheral circadian clocks by high-throughput chemical screening. Proc. Natl Acad. Sci. USA.

[35] Gloston GF, Yoo SH, Chen ZJ (2017). Clock-enhancing small molecules and potential applications in chronic diseases and aging. Front Neurol..

[36] He B (2016). The small molecule nobiletin targets the molecular oscillator to enhance circadian rhythms and protect against metabolic syndrome. Cell Metab..

[37] Nohara K (2015). Ammonia-lowering activities and carbamoyl phosphate synthetase 1 (Cps1) induction mechanism of a natural flavonoid. Nutr. Metab. (Lond.).

[38] Koike N (2012). Transcriptional architecture and chromatin landscape of the core circadian clock in mammals. Science.

[39] Takeda Y (2014). Retinoic acid-related orphan receptor gamma (RORgamma): a novel participant in the diurnal regulation of hepatic gluconeogenesis and insulin sensitivity. PLoS Genet..

[40] Enriquez JA (2016). Supramolecular organization of respiratory complexes. Annu. Rev. Physiol..

[41] Jha P, Wang X, Auwerx J (2016). Analysis of mitochondrial respiratory chain supercomplexes using blue native polyacrylamide gel electrophoresis (BN-PAGE). Curr. Protoc. Mouse Biol..

[42] Mourier A, Matic S, Ruzzenente B, Larsson NG, Milenkovic D (2014). The respiratory chain supercomplex organization is independent of COX7a2l isoforms. Cell Metab..

[43] Cogliati S (2016). Mechanism of super-assembly of respiratory complexes III and IV. Nature.

[44] Guo R, Zong S, Wu M, Gu J, Yang M (2017). Architecture of human mitochondrial respiratory megacomplex I2III2IV2. Cell.

[45] Bultema JB, Braun HP, Boekema EJ, Kouril R (2009). Megacomplex organization of the oxidative phosphorylation system by structural analysis of respiratory supercomplexes from potato. Biochim. Biophys. Acta.

[46] Shinozaki A (2017). Potent effects of flavonoid nobiletin on amplitude, period, and phase of the circadian clock rhythm in PER2::LUCIFERASE mouse embryonic fibroblasts. PLoS ONE.

[47] Woldt E (2013). Rev-erb-alpha modulates skeletal muscle oxidative capacity by regulating mitochondrial biogenesis and autophagy. Nat. Med..

[48] Dyar KA (2014). Muscle insulin sensitivity and glucose metabolism are controlled by the intrinsic muscle clock. Mol. Metab..

[49] Raichur S (2010). Identification and validation of the pathways and functions regulated by the orphan nuclear receptor, ROR alpha1, in skeletal muscle. Nucleic Acids Res..

[50] Acin-Perez R (2004). Respiratory complex III is required to maintain complex I in mammalian mitochondria. Mol. Cell.

[51] Maranzana E, Barbero G, Falasca AI, Lenaz G, Genova ML (2013). Mitochondrial respiratory supercomplex association limits production of reactive oxygen species from complex I. Antioxid. Redox Signal..

[52] Williams EG (2016). Systems proteomics of liver mitochondria function. Science.

[53] Wyse CA, Coogan AN (2010). Impact of aging on diurnal expression patterns of CLOCK and BMAL1 in the mouse brain. Brain Res..

[54] Tulsian R, Velingkaar N, Kondratov R (2018). Caloric restriction effects on liver mTOR signaling are time-of-day dependent. Aging (Albany NY).

[55] Masri S (2014). Partitioning circadian transcription by SIRT6 leads to segregated control of cellular metabolism. Cell.

[56] Peek CB (2013). Circadian clock NAD+ cycle drives mitochondrial oxidative metabolism in mice. Science.

[57] Miller RA (2007). An aging interventions testing program: study design and interim report. Aging Cell.

[58] Harrison DE (2009). Rapamycin fed late in life extends lifespan in genetically heterogeneous mice. Nature.

[59] Strong R (2016). Longer lifespan in male mice treated with a weakly estrogenic agonist, an antioxidant, an alpha-glucosidase inhibitor or a Nrf2-inducer. Aging Cell.

[60] Brandhorst S (2015). A periodic diet that mimics fasting promotes multi-system regeneration, enhanced cognitive performance, and healthspan. Cell Metab..

[61] Newman JC (2017). Ketogenic diet reduces midlife mortality and improves memory in aging mice. Cell Metab..

[62] Charles KN (2017). Uncoupling of metabolic health from longevity through genetic alteration of adipose tissue lipid-binding proteins. Cell Rep..

[63] Levine ME (2014). Low protein intake is associated with a major reduction in IGF-1, cancer, and overall mortality in the 65 and younger but not older population. Cell Metab..

[64] Sanjana NE, Shalem O, Zhang F (2014). Improved vectors and genome-wide libraries for CRISPR screening. Nat. Methods.

[65] Trapnell C, Pachter L, Salzberg SL (2009). TopHat: discovering splice junctions with RNA-Seq. Bioinformatics.

[66] Trapnell C (2010). Transcript assembly and quantification by RNA-Seq reveals unannotated transcripts and isoform switching during cell differentiation. Nat. Biotechnol..

[67] Nemkov T, Hansen KC, D’Alessandro A (2017). A three-minute method for high-throughput quantitative metabolomics and quantitative tracing experiments of central carbon and nitrogen pathways. Rapid Commun. Mass Spectrom..

[68] Clasquin M. F., Melamud E., Rabinowitz J. D. LC-MS data processing with MAVEN: a metabolomic analysis and visualization engine. *Curr. Protoc. Bioinformatics* Chapter 14, Unit14 11 (2012). 10.1002/0471250953.bi1411s37 PMC4055029 22389014

[69] Mileykovskaya E (2012). Arrangement of the respiratory chain complexes in Saccharomyces cerevisiae supercomplex III2IV2 revealed by single particle cryo-electron microscopy. J. Biol. Chem..

[70] Chida J, Yamane K, Takei T, Kido H (2012). An efficient extraction method for quantitation of adenosine triphosphate in mammalian tissues and cells. Anal. Chim. Acta.

