# Supplementary material for: Nobiletin fortifies mitochondrial respiration in skeletal muscle to promote healthy aging against metabolic challenge
Source: Nat Commun. 2019 Aug 28;10:3923. doi: 10.1038/s41467-019-11926-y (PMC6713763; doi:10.1038/s41467-019-11926-y)
Supplement: Supplementary file 1 — Supplementary Information [file 41467_2019_11926_MOESM1_ESM.pdf]

Supplementary Information

for

Nobiletin fortifies mitochondrial respiration in skeletal muscle to promote healthy aging against metabolic challenge

Nohara et al.

Supplementary Figure 1

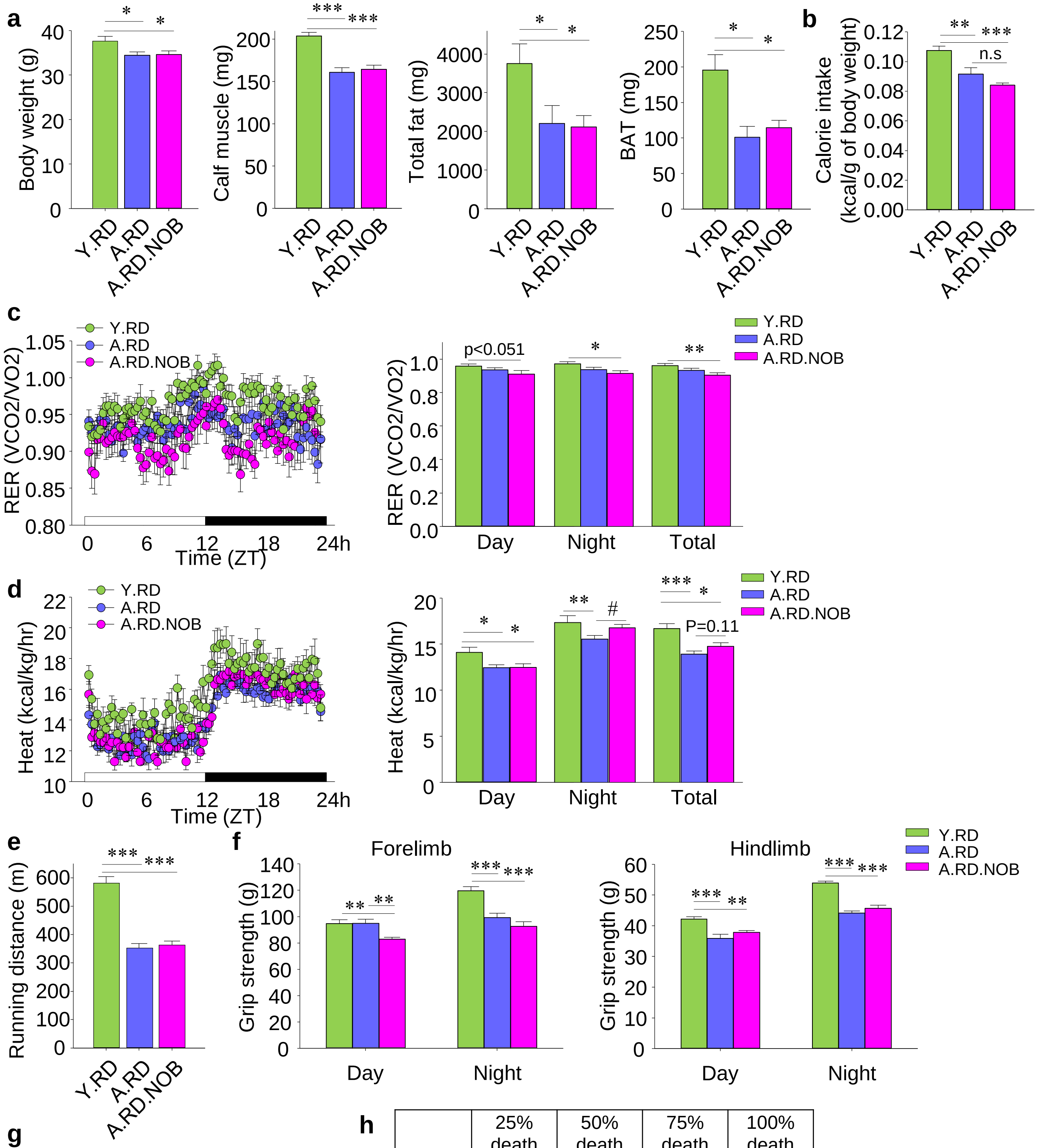

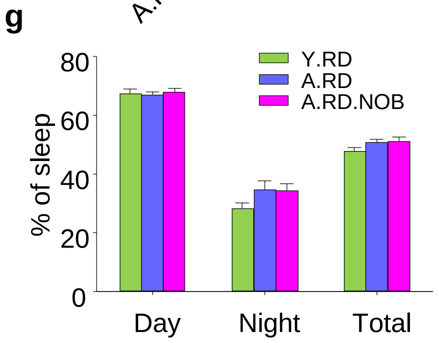

h

	25% death	50% death	75% death	100% death
RD (Days)	791.25	884.5	965	1040
RD.NOB (Days)	862	921	944.5	1083
Log-rank test				
p values	0.12	**0.016***	0.47	0.76
Mann-Whitney U test				
p values	0.118	**0.042***	0.191	0.448

Supplementary Figure 1. NOB improved metabolic profile and midlife survival in regular diet (RD) feeding. **a** End-point body weight and tissue weight after 20 weeks of treatment (n=10, 5 and 7 for Y.RD, A.RD and A.RD.NOB respectively). **b** Daily caloric intake was calculated with normalization for body weight (n=10, 7 and 10 for Y.RD, A.RD and A.RD.NOB). **c** In vivo respiration exchange rate (RER) and **d** heat production measured in metabolic chamber. Average values of day, night and total are shown (n=9, 10 and 9 for Y.RD, A.RD and A.RD.NOB). **e** Running distances were measured with treadmill (n=10, 8 and 9 for Y.RD, A.RD and A.RD.NOB). **f** Grip strength test (ZT6, n=10, 9 and 9 for Y.RD, A.RD and A.RD.NOB; ZT18, n=10, 9 and 9 for Y.RD, A.RD and A.RD.NOB). **g** Percent of sleep was analyzed with the PIEZO sleep analysis system (n=9, 5 and 8 for Y.RD, A.RD and A.RD.NOB). * $p<0.05$, ** $p<0.01$, *** $p<0.001$, one-Way ANOVA; # $p<0.05$, t-test. Data are presented as mean ± SEM in bar and line graphs. **h** Quantile analysis of survival data in **Fig 1e** (n=20). P values are calculated by Log-rank and Mann-Whitney U tests.

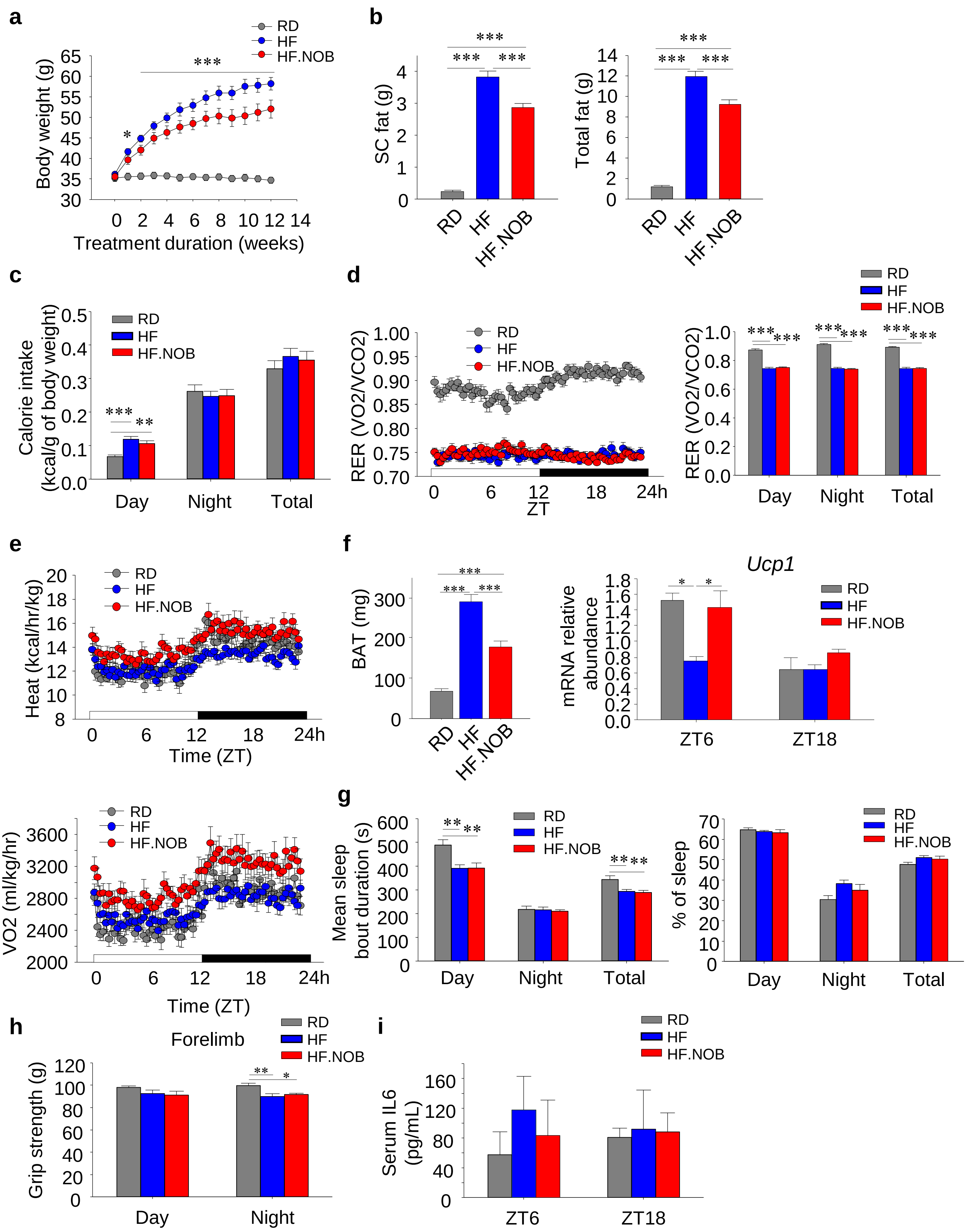
a
Body weight (g)
Treatment duration (weeks)
RD
HF
HF.NOB
b
SC fat (g)
Total fat (g)
c
Calorie intake (kcal/g of body weight)
Day
Night
Total
d
RER (VO2/VCO2)
ZT
e
Heat (kcal/hr/kg)
Time (ZT)
VO2 (ml/kg/hr)
f
BAT (mg)
Ucp1
mRNA relative abundance
ZT6
ZT18
g
Mean sleep bout duration (s)
% of sleep
h
Forelimb
Grip strength (g)
i
Serum IL6 (pg/mL)

Supplementary Figure 2. NOB alleviated adiposity and improved glucose homeostasis in high-fat (HF) feeding. **a** Body weight during 12 weeks of treatment (n=12, 11 and 13 for RD, HF and HF.NOB respectively). **b** Subcutaneous (SC) fat mass and total fat weight were measured (n=20, 16 and 17 for RD, HF and HF.NOB). **c** Daily caloric intake was measured through 7 days. Daily average values are shown (n=6, 7 and 6 for RD, HF and HF.NOB). **d** In vivo respiration exchange rate (RER). Average values of day, night and total are shown (n=9, 10 and 10 for RD, HF and HF.NOB). **e** In vivo heat production (upper panel) and oxygen consumption (lower panel) related to Fig 2e (n=9, 10 and 10 for RD, HF and HF.NOB). **f** Left panel: Brown adipose tissue (BAT) weight after 20 weeks of treatment in the HF feeding condition (n=20, 16 and 17 for RD, HF and HF.NOB). Right panel: *Ucp1* gene expression levels in BAT (ZT6, n=3, 4 and 5 for RD, HF and HF.NOB; ZT18, n=6, 6 and 4 for RD, HF and HF.NOB). **g** Mean sleep bout duration and % of sleep was analyzed with Piezo sleep analysis system (n=8, 10 and 13 for RD, HF and HF.NOB). **h** Forelimb grip strength test (n=5, 6 and 8 for RD, HF and HF.NOB). **i** Serum IL-6 levels (ZT6, n=5, 5 and 6 for RD, HF and HF.NOB; ZT18, n=5, 7 and 5 for RD, HF and HF.NOB). * $p<0.05$, ** $p<0.01$, *** $p<0.001$, One-Way ANOVA. Data are presented as mean ±SEM in bar and line graphs.

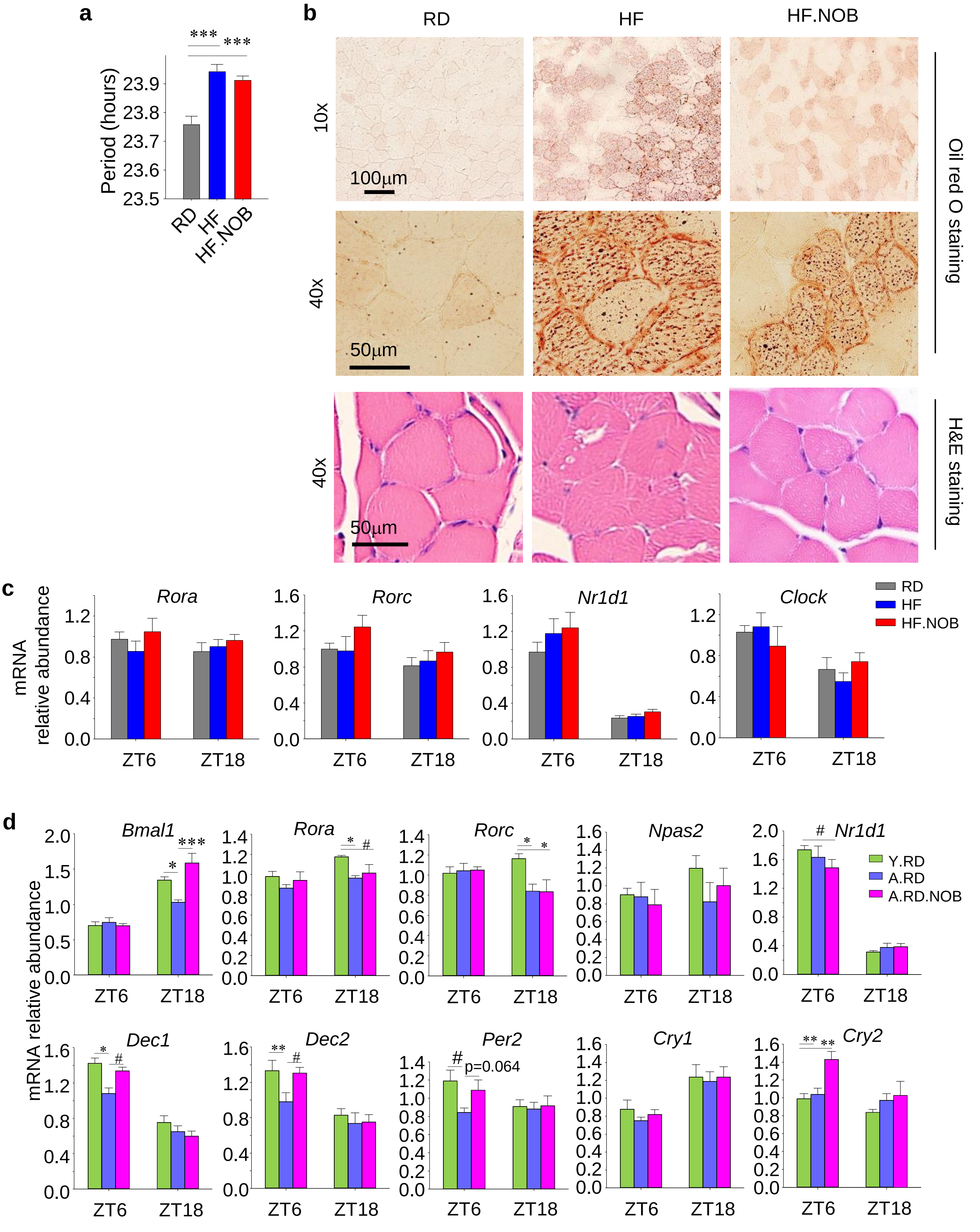
a
Period (hours)
RD
HF
HF.NOB
b
RD
HF
HF.NOB
10x
40x
100μm
50μm
Oil red O staining
H&E staining
c
mRNA relative abundance
Rora
Rorc
Nr1d1
Clock
ZT6
ZT18
d
Bmal1
Npas2
Dec1
Dec2
Per2
Cry1
Cry2
Y.RD
A.RD
A.RD.NOB
p=0.064

Supplementary Figure 3. NOB diminished lipid accumulation in skeletal muscle. **a** Circadian period lengths were calculated under constant darkness by using the ActiView software (n=7, 6 and 6 for RD, HF and HF.NOB). **b** Calf muscle tissues from RD, HF and HF.NOB groups were stained by Oil red O (top and middle rows) and H&E (bottom row). Representative pictures are shown. (n=6, 6 and 4 for RD, HF and HF.NOB). **c** Expression of *Rora*, *Rorc* and *Nr1d1*, and *Clock* genes in the HF condition (ZT6, n=11, 10 and 10 for RD, HF and HF.NOB; ZT18, n=9, 8 and 8 for RD, HF and HF.NOB). **d** Expression of clock genes in the RD condition (ZT6, n=10, 6 and 5 for Y.RD, A.RD and A.RD.NOB; ZT18, n=10, 6 and 6 for Y.RD, A.RD and A.RD.NOB). * p<0.05, ** p<0.01, *** p<0.001, One-Way ANOVA, # p<0.05, t-test. Data are presented as mean ±SEM in bar graphs.

Supplementary Figure 4

a

Top Canonical Pathways
EIF2 Signaling
**Mitochondrial Dysfunction**
**Oxidative Phosphorylation**
mTOR Signaling
Regulation of eIF4, p70S6K signaling

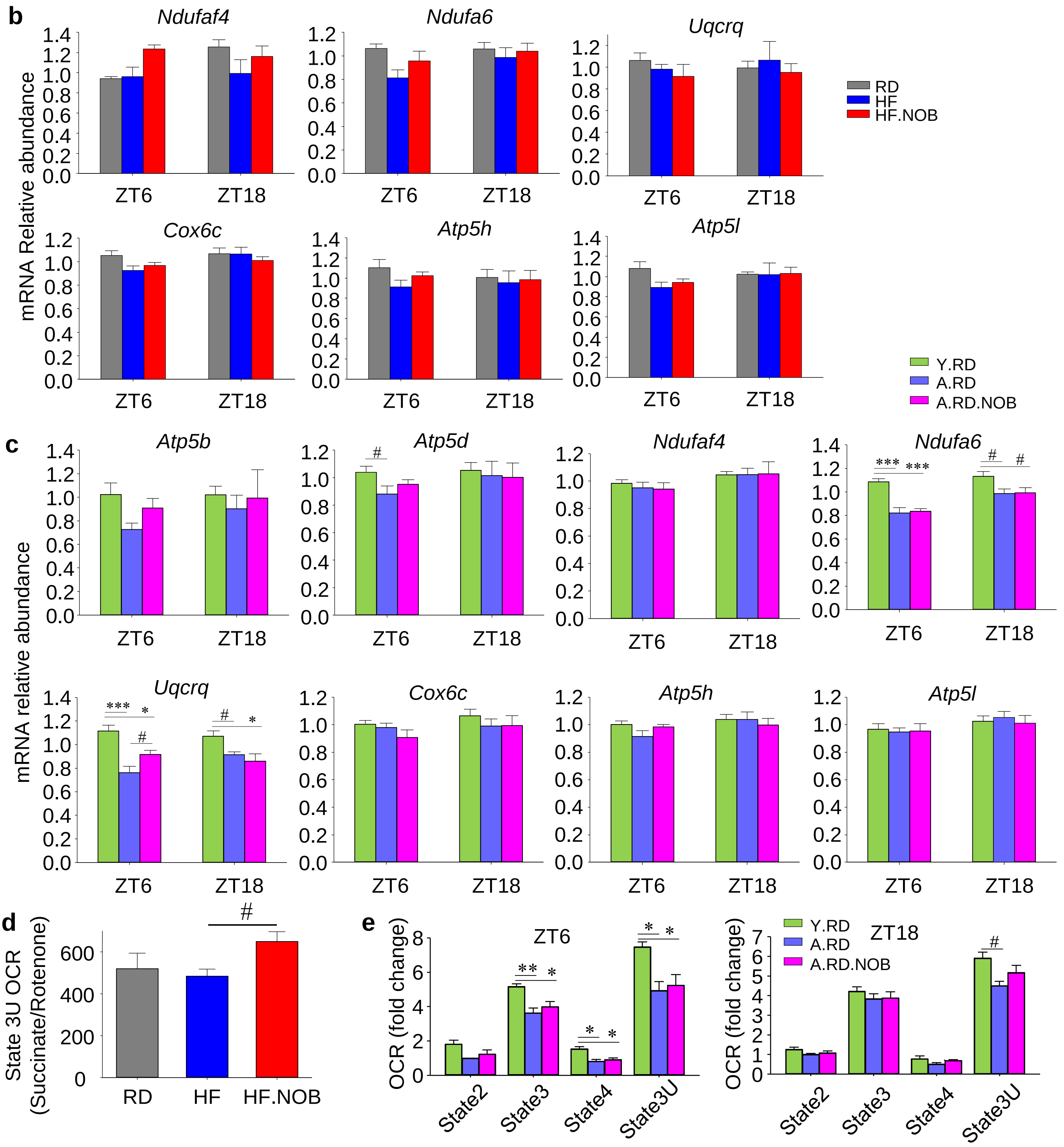

Supplementary Figure 4. NOB activated mitochondrial OXPHOS gene expression. **a** Top 5 canonical pathways. RNA sequencing data were analyzed by using the IPA software. Two mitochondrial function related pathways were identified as the top pathways in HF vs HF.NOB at ZT6. **b** qPCR analysis results for expression of Ndufaf4 and Ndufa6 (both Complex I), Uqcrq (Complex III), Cox6c (Complex IV), Atp5h and Atp5l (Complex V) are shown (ZT6, n=5, 5 and 6 for RD, HF and HF.NOB; ZT18, n=6, 6 and 4 for RD, HF and HF.NOB). **c** Mitochondrial gene expression (ZT6, n=10, 6 and 5 for Y.RD, A.RD and A.RD.NOB; ZT18, n=10, 6 and 6 for Y.RD, A.RD and A.RD.NOB). **d** State 3U Oxygen consumption rate (OCR) in mitochondria isolated from aged calf muscle under the HF feeding condition. Seahorse analysis was performed with rotenone treatment followed by succinate (ZT6, n=3, 4 and 4 for RD, HF and HF.NOB). **e** OCR in mitochondria isolated from aged calf muscle in the RD feeding condition. Treatment was the same as described for Fig 4c (ZT6, n=5, 3 and 5 for Y.RD, A.RD and A.RD.NOB; ZT18, n=5, 4 and 5 for Y.RD, A.RD and A.RD.NOB). * $p<0.05$, *** $p<0.001$, One-Way ANOVA, # $p<0.05$, t-test. Data are presented as mean ±SEM in bar graphs.

Supplementary Figure 5

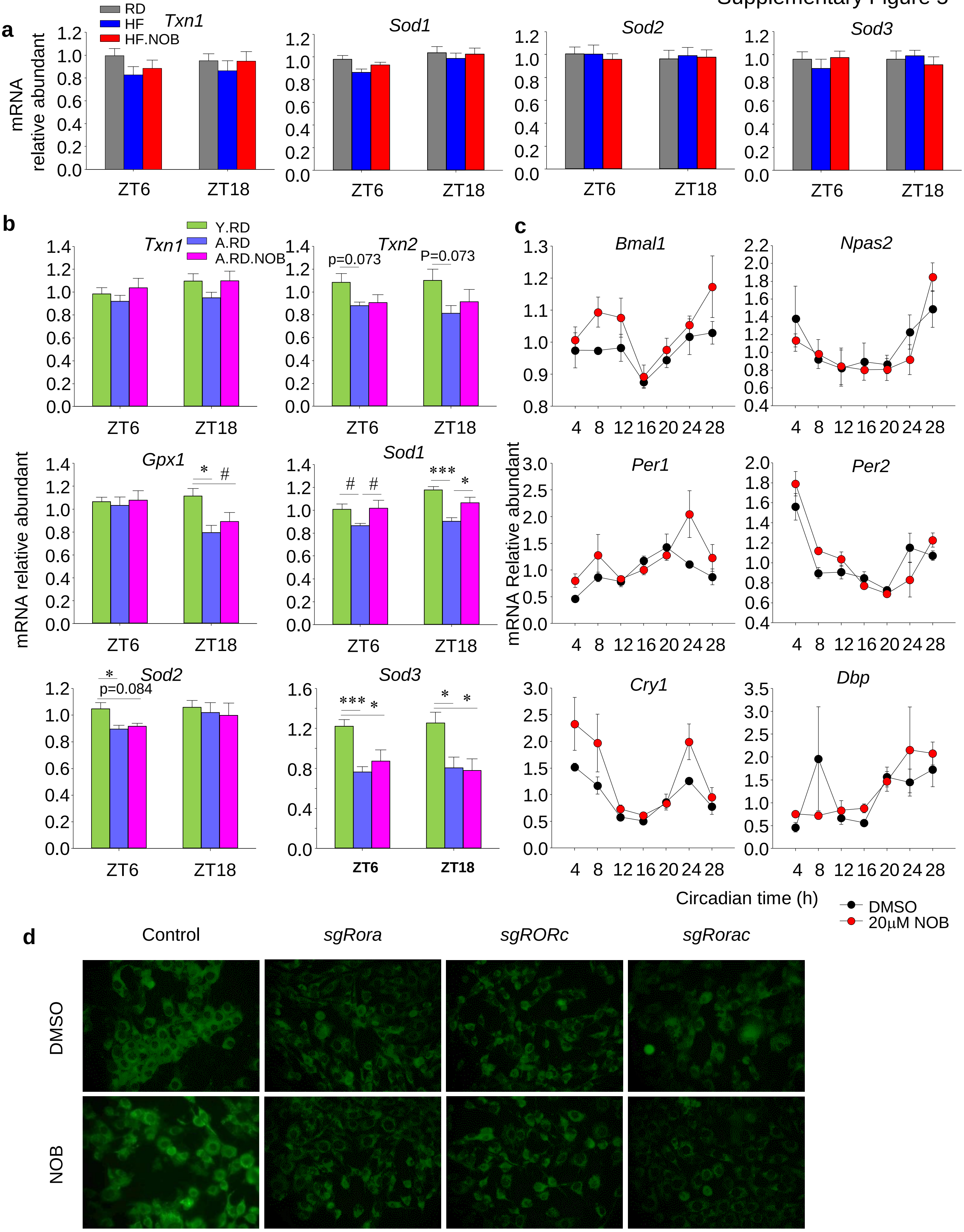

Supplementary Figure 5. Effects of NOB on redox gene expression. **a** Real-time qPCR analysis of gene expression for the anti-oxidants Txn1 and SODs (ZT6, n=11, 10 and 9 for RD, HF and HF.NOB; ZT18, n=10, 11 and 10 for RD, HF and HF.NOB). **b** Real-time qPCR analysis of redox gene expression (ZT6, n=10, 6 and 5 for Y.RD, A.RD and A.RD.NOB; ZT18, n=10, 6 and 6 for Y.RD, A.RD and A.RD.NOB). (**a** and **b**) * $p<0.05$, *** $p<0.001$, One-Way ANOVA, # $p<0.05$, t-test. **c** Real-time qPCR analysis of clock gene expression in differentiated C2C12 myotubes with or without NOB treatment (n=3). Two-Way ANOVA showed statistically significant differences between DMSO and NOB for *Bmal1* ($p<0.05$), *Cry1* ($p<0.01$), *Per1* (0.01). **d** MitoTracker deep red staining. Grayscale MitoTracker images from a Leica Inverted Phase Contrast Microscope were re-colored to green using associated software. Representative images are shown. Data are presented as mean ±SEM in bar and line graphs.

Supplementary Figure 6

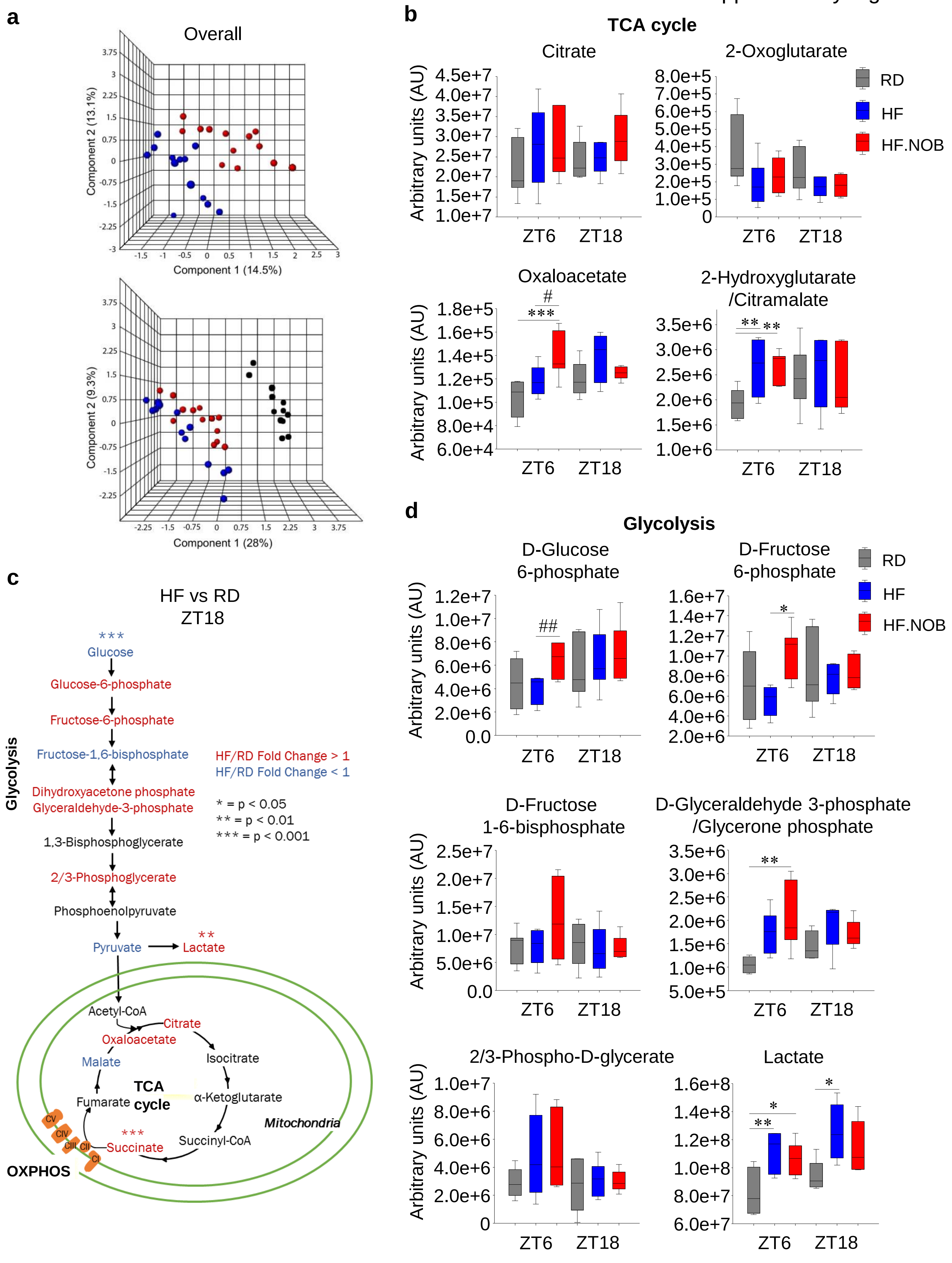

Supplementary Figure 6. NOB regulates TCA and glycolysis flux. Aged skeletal muscle tissues were subjected to unbiased metabolomic analysis. **a** PLS-DA analysis results are 3D-plotted for overall. Black, blue and red dots indicate individual RD. HF and HF.NOB treated mice (n=14, 13 and 14 for RD, HF and HF.NOB). **b** TCA cycle metabolites in skeletal muscle (ZT6, n=7, 7 and 8 for RD, HF and HF.NOB; ZT18, n=7, 7 and 6 for RD, HF and HF.NOB). **c** Glycolysis and TCA pathways between RD and HF in skeletal muscle at ZT18. Red and blue indicate fold change ≥ 1.1 and ≤ 0.9 respectively. n=7, 7 and 6 for RD, HF and HF.NOB). **d** Glycolysis related metabolites in skeletal muscle (ZT6, n=7, 7 and 8 for RD, HF and HF.NOB; ZT18, n=7, 7 and 6 for RD, HF and HF.NOB). * p<0.05, ** p<0.01, *** p<0.001, one-Way ANOVA; # p<0.05, ## p<0.01, t-test. Box-whisker plots, box edges correspond to 25th and 75th percentiles, lines inside of box correspond 50th percentiles and whiskers include extreme data points.

Supplementary Figure 7

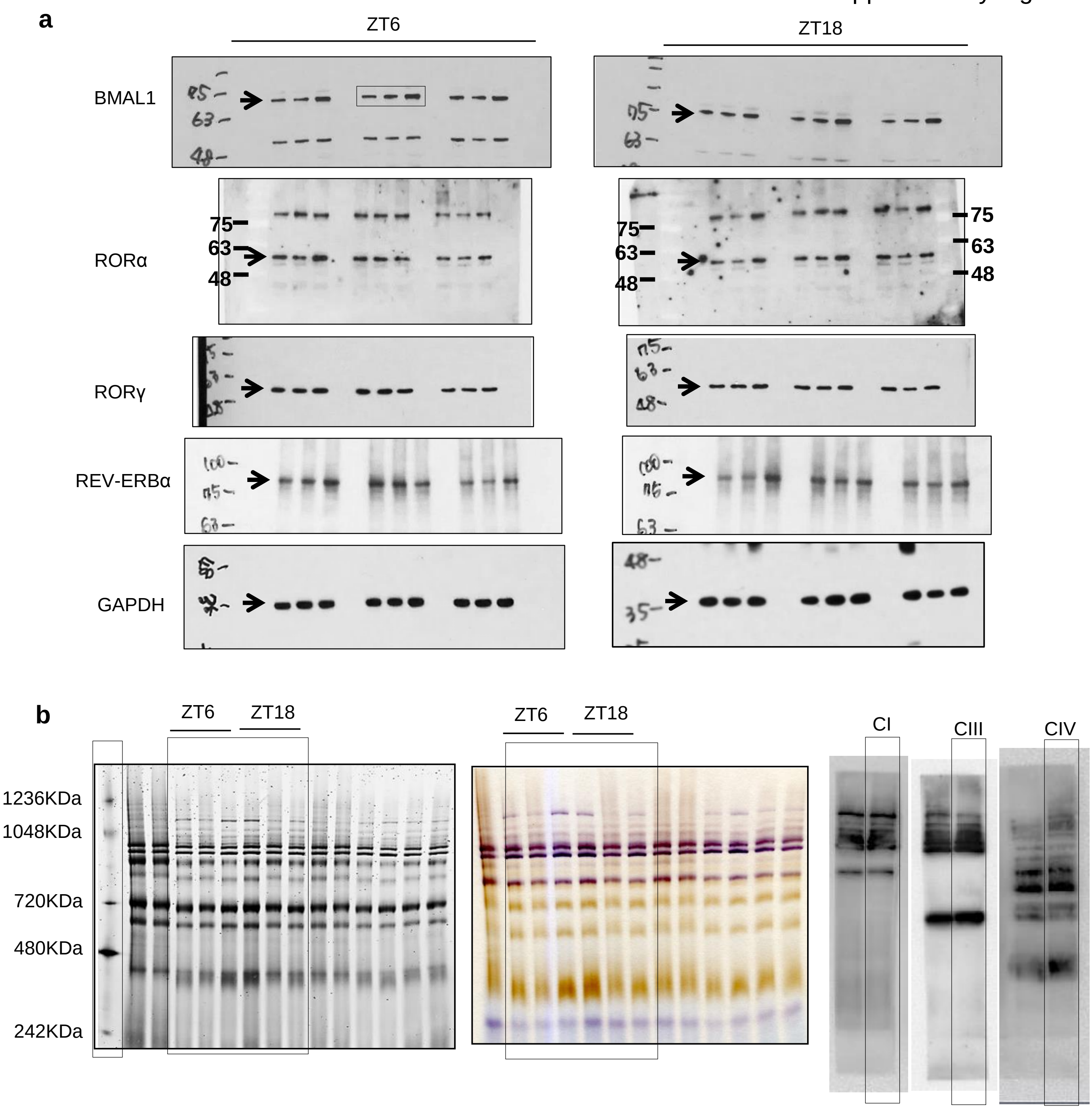

Supplementary Figure 7. Supporting Western blot images for Fig 3 and Fig 7. **a** Uncropped images for Western blots in Fig 3g. **b** Uncropped images for Fig 7a and 7b (left), Fig 7c (middle) and Fig 7d (right).

Supplementary Table 1: Sense (forward) and anti-sense (reverse) gRNAs for Rora and Rorc CRIPSR.

**gRNA target**	**Sequence**
Rora-1f	caccgAGAGACAGCTTGTACGCCG
Rora-1r	aaacCGGCGTACAAGCTGTCTCTc
Rora-2f	caccgACCTCAGCACCTATATGGA
Rora-2r	aaacTCCATATAGGTGCTGAGGTc
Rora-3f	caccgAAACCGCTGCCAGCATTGT
Rora-3r	aaacACAATGCTGGCAGCGGTTTc
Rorc-1f	caccgTGATCCCTTGCAAGATCTG
Rorc-1r	aaacCAGATCTTGCAAGGGATCAc
Rorc-2f	caccgCAGCAGCAACAGGAACAAG
Rorc-2r	aaacCTTGTTCCTGTTGCTGCTGc
Rorc-3f	caccgACAGCATCTATAGCACTGA
Rorc-3r	aaacTCAGTGCTATAGATGCTGTc

Supplementary Table 2: qPCR primers.

Target gene	Forward primer	Reverse primer
Actb	TTGTCCCCCCAACTTGATGT	CCTGGCTGCCTCAACACCT
Bmal1	CCAAGAAAGTATGGACACAGACAAA	GCATTCTTGATCCTTCCTTGGT
Npas2	CAACAGACGGCAGCATCATCT	TTCTGATCCATGACATCCGC
Dec1	GCAAGGAAACTTACAAACTGCC	CAATGCACTCGTTAATCCGGT
Dec2	ATTGCTTTACAGAATGGGGAGCG	AAAGCGCGCGAGGTATTGCAAGAC
Rora	GCACCTGACCGAAGACGAAA	GAGCGATCCGCTGACATCA
Rorc	TCAGCGCCCTGTGTTTTTC	GAGAACCAGGGCCGTGTAG
Nr1d1	CATGGTGCTACTGTGTAAGGTGTGT	CACAGGCGTGCACTCCATAG
Clock	CCTTCAGCAGTCAGTCCATAAAC	AGACATCGCTGGCTGTGTTAA
Gapdh	CAAGGTCATCCATGACAACTTTG	GGCCATCCACAGTCTTCTGG
Ndufaf4	CCGGAGTCAGTATCCAGAAATC	CCTTTGGTTCTTGTCGTGGTTC
Ndufa6	CGGTGAAGCCCATTTTCAGTC	GCATTAAGTGCACGGTGTTCG
Uqcrq	TTCAGCAAAGGCATCCCCAA	TAGACCACTACAAACGGCGG
Cox6c	GCTGCCTATAAGTTTGGCGTG	GCACTCTGAAAGATACCAGCC
Atp5b	GGTTCATCCTGCCAGAGACTA	AATCCCTCATCGAACTGGACG
Atp5d	TGCTTCAGGCGCGTACATAC	CACTTGCTTGACGTTGGCA
Atp5h	GCTGGGCGTAAACTTGCTCTA	CAGACAGACTAGCCAACCTGG
Atp5l	GAGAAGGCACCGTCGATGG	ACACTCTGAATAGCTGTAGGGAT
Ucp1	AGGCTTCCAGTACCATTAGGT	CTGAGTGAGGCAAAGCTGATTT
Gpx1	CACCGAGATGAACGATCTGC	CATTCTCCTGGTGTCCGAACTG
Tnx1	CATGCCGACCTTCCAGTTTTA	TTTCCTTGTTAGCACCGGAGA
Tnx2	TGGGCTTCCCTCACCTCTAAG	CCTGGACGTTAAAGGTCGTCA
Sod1	AACCAGTTGTGTTGTCAGGAC	CCACCATGTTTCTTAGAGTGAGG
Sod2	CAGACCTGCCTTACGACTATGG	CTCGGTGGCGTTGAGATTGTT
Sod3	CCTTCTTGTTCTACGGCTTGC	TCGCCTATCTTCTCAACCAGG

Supplementary Methods

Metabolomic analysis

Sample Preparation: Isolated calf muscle tissue was flash frozen in liquid nitrogen and stored at -80°C until analysis. Prior to LC-MS analysis, samples were placed on ice and suspended with methanol:acetonitrile:water (5:3:2, v:v) to a concentration of 30 mg/ml. Glass beads (GB10, Next Advance, Troy, NY, USA) were added to each tube and placed into a Bullet Blender (Next Advance, Troy, NY, USA) at setting 3 for 5 min at 4°C to homogenize tissue. Suspensions were then vortexed continuously for 30 min at 4°C. Insoluble material was removed by centrifugation at 10,000 g for 10 min at 4°C and supernatants were isolated for metabolomics analysis by UHPLC-MS.

UHPLC-MS analysis: Analyses were performed as previously published [1]. Briefly, the analytical platform employs a Vanquish UHPLC system (ThermoFisher Scientific, San Jose, CA, USA) coupled online to a Q Exactive mass spectrometer (Thermo Fisher Scientific, San Jose, CA, USA). Samples were resolved over a Kinetex C18 column, 2.1 x 150 mm, 1.7 µm particle size (Phenomenex, Torrance, CA, USA) equipped with a guard column (SecurityGuard™ Ultracartridge – UHPLC C18 for 2.1 mm ID Columns – AJO-8782 – Phenomenex, Torrance, CA, USA) using an aqueous phase (A) of water and 0.1% formic acid and a mobile phase (B) of acetonitrile and 0.1% formic acid. Samples were eluted from the column using either an isocratic elution of 5% B flowed at 250 µl/min and 25ºC or a gradient from 5% to 95% B over 1 minute, followed by an isocratic hold at 95% B for 2 minutes, flowed at 400 µl/min and 30ºC. The Q Exactive mass spectrometer (Thermo Fisher Scientific, San Jose, CA, USA) was operated independently in positive or negative ion mode, scanning in Full MS mode (2 µscans) from 60 to 900 m/z at 70,000 resolution, with 4 kV spray voltage, 15 shealth gas, 5 auxiliary gas. Calibration was performed prior to analysis using the Pierce™ Positive and Negative Ion Calibration Solutions

(Thermo Fisher Scientific). Acquired data was then converted from .raw to .mzXML file format using Mass Matrix (Cleveland, OH, USA). Samples were analyzed in randomized order with a technical mixture injected after every 15 samples to qualify instrument performance. Metabolite assignments, isotopologue distributions, and correction for expected natural abundances of deuterium, ^{13}C, and ^{15}N isotopes were performed using MAVEN (Princeton, NJ, USA) [2].

Graphs, heat maps and statistical analyses (either T-Test or ANOVA), metabolic pathway analysis, PLS-DA and hierarchical clustering was performed using the MetaboAnalyst 3.0 package (www.metaboanalyst.com) [3]. Hierarchical clustering analysis (HCA) was also performed through the software GENE-E (Broad Institute, Cambridge, MA, USA). XY graphs were plotted through GraphPad Prism 5.0 (GraphPad Software Inc., La Jolla, CA, USA).

Mitochondria isolation and biochemical analyses

Mitochondria isolation from calf muscle

Mitochondrial fractions were isolated from fresh calf muscle by the Potter-Elvehjem method [4, 5]. In brief, Isolated calf muscle tissues were resuspended in ice-cold basic media [BM; 140mM KCl, 20mM HEPES, 5mM MgCl2, 1mM EGTA, 10mM pyruvate, and 2mM malate (pH 7.0)], minced in homogenization medium (BM containing 1mM ATP, 11U/mg subtilisin A and 0.1% BSA) and homogenized with a Potter-Elvehjem homogenizer on ice. Following centrifugation at 1000g at 4ºC for 10min, supernatants were further centrifuged at 8000g at 4ºC for 10min. The mitochondria pellets were resuspended in BM and allowed to sit on ice for 5 min to induce myofibril repolymerization. The samples were centrifuged at 800g at 4ºC for 10min to remove myofibers. The supernatants were centrifuged at 8000g at 4ºC for 10min, and the resultant pellet was resuspended in 200µL of ice-cold BM with gently pipetting. Protein content was determined by Bradford assays.

Solubilization of mitochondria

Purified mitochondria were solubilized in digitonin according to the procedure [6] with modifications. Isolated mitochondria were suspended in lysis buffer containing 2% (w/v) digitonin (Sigma) (8g/g, digitonin-protein ratio), 50mM potassium acetate, 10% glycerol, 1X complete Mini EDTA-free Protease Inhibitor Cocktail (Roche), and 30 mM HEPES-KOH (pH 7.4) for 1 h at 4 °C with gentle shaking. After incubation the lysate was centrifuged at 4 °C for 20 min at 45,000 rpm (TLA55 rotor). Supernatant containing lysates were analyzed for protein estimation, Blue-Native PAGE, and Western blotting.

To extract the respiratory SCs, mitochondria purified from the muscle tissue were solubilized by mild detergent digitonin (8g/g of protein). Usual ratios are in the range from 4g/g to 8g/g [7, 8, 9]; for the aged mice 8g/g was the best treatment [10].

After digitonin extraction, the respiratory chain complexes and SCs were separated by BN-PAGE and subjected to in-gel enzyme activity staining or Western blot analysis with antibodies against subunits of Complexes I, III and IV.

BN-PAGE for analysis of supercomplexes

Mitochondrial lysates were subjected to BN-PAGE [8] in a 3–12% acrylamide gradient gel in Bis-Tris buffer (Invitrogen) according to manufacturer’s instructions. After electrophoresis gels were either stained with Imperial stain (Thermo Fisher Scientific) and Bio-Safe Coomassie G-250 (Bio-Rad) or used for Western blotting analysis.

In-gel enzyme activity

For CI and CIV in-gel activity staining, mitochondrial lysates were subjected to BN-PAGE [7] with modifications. Samples were loaded onto a 3-12% Bis-Tris gradient gel and electrophoresed for 30 min at constant 250V at 12-13 mA. After 30 min, dark blue cathode buffer in the inner chamber was changed to light blue cathode buffer, and the gel was further electrophoresed for 6h at low

amplitude (6-7 mA). After BN-PAGE, the gels were washed at least 2 times with water and subjected to in-gel activity staining.

In-gel activity assays were performed as described [7] with minor modifications.
CI (NADH:dehydrogenase) in-gel activity: Gels were incubated for 20 min in buffer containing 250 mM Tris, pH 7.4, 140 µM NADH, and 3 mM Nitrotetrazolium Blue chloride (NBT). Appearance of violet bands is indicative of complex I activity. Reaction was stopped by adding 10% acetic acid.

CIV (cytochrome c oxidase) in-gel activity: Gels were incubated in 50mM phosphate buffer, pH 7.4, 0.5 mg/ml diaminobenzidine (DAB), and 1mg/ml bovine heart cytochrome c. At least 20 min incubation was required to see presence of brown bands indicative for CIV activity. The gels were either immediately used for CI in-gel activity staining or reaction was stopped by adding 10% acetic acid.

Seahorse mitochondrial oxygen consumption rate (OCR) analysis

Isolated mitochondria were applied to Seahorse Bioanalyzer to determine bioenergetic flux. In brief, 5 µg of mitochondrial protein/well was seeded onto an XF-24 V7 plate and centrifuged at 2000g at 4ºC for 20min to attach mitochondrion onto the plate. Reaction medium [RM; 70 mM sucrose, 220 mM mannitol, 2 mM HEPES, 1 mM EGTA, 10mM pyruvate, 2 mM malate, 0.2% BSA, 10 mM KH2PO4 (pH 7.2)] was added to incubate at 37ºC for 10min prior to Seahorse run. After basal OCR measurement, mitochondria were treated with ADP (final conc. 4 mM), oligomycin (final conc. 2.5mg/mL), FCCP (final conc. 4uM) and Antimycin A (final conc. 4 uM). For C2C12 culture cells, cells were seeded on a V7 assay plate and maintained until ~90% confluency. Differentiation was performed as above. At Day 5, cells were pre-treated with 20 µM Nobiletin in differentiation media for 24 hrs. On the day of the Seahorse assay, differentiation media was removed and cells were rinsed with Seahorse assay media containing 10 mM D-Glucose and 10 mM Sodium Pyruvate with vehicle or 20 µM NOB. Cells were further incubated

in assay media in non-CO2 incubator at 37ºC for 1 hr. After basal OCR measurement, cells were treated with vehicle or tBHP (Port A; final conc. 1mM), oligomycin (Port B; final conc. 2.5 mg/mL), FCCP (Port C; final conc. 4 µM) and Antimycin A (Port D; final conc. 2 µM). In the rotenone/succinate experiments, after basal OCR measurement, cells were treated with Rotenone (Port A; final conc. 2 µM), Succinate (Port B; final conc. 10 mM) and Antimycin A (Port D; final conc. 2 µM) in the presence of 4 µM FCCP.

For Rora and Rorc individual and double knockdown ((RoraKD, RorcKD, and RoracKD) C2C12 cells, cells were seeded on a V7 assay plate and maintained for 24 hrs. Cells were then pre-treated with 20µM NOB in culture media for 24 hrs. On the day of the Seahorse assay, differentiation media was removed and rinsed with Seahorse assay media containing 10 mM D-Glucose and 10mM Sodium Pyruvate with vehicle or 20µM NOB. Cells were further incubated in assay media in non-CO2 incubator at 37 ºC for 1 hr to equilibrate. After basal OCR measurement, cells were treated with vehicle or tBHP (Port A; final conc. 0.25 mM), oligomycin (Port B; final conc. 2.5 mg/mL), FCCP (Port C; final conc. 4 µM) and Antimycin A (Port D; final conc. 2 µM).

Western blotting

Western blotting was performed using established methods [11]. In brief, calf muscle was isolated at ZT6 and ZT18, and snap frozen in liquid nitrogen. Piece of tissue was homogenized and protein lysate was extracted after centrifugation. Five to ten microgram of protein samples were loaded and run onto SDS-PAGE followed by transferring onto nitrocellulose membrane by semi-dry transfer system. After blocking with blocking buffer, membranes were incubated with primary antibodies; BMAL1 (epitope: aa 1-230, Cocalico Biologicals) and IgY were affinity purified using the same protein, 1:4000), RORα (epitope: aa 105-122, Cocalico Biologicals, 1:1000), RORγ (epitope: aa 212-230, Cocalico Biologicals; 1:1000), REV-ERBα (Millipore; 1:1000) and GAPDH (Santa Cruz SC25778; 1:5000). Horse radish peroxidase (HRP) conjugated anti-Rabbit, anti-

mouse, anti-chicken and anti-guinea pig secondary antibodies were used as appropriate with 1:1000-1:10000 dilution. Signals were detected by ECL.

Western blotting for the detection of the respiratory complexes CI, CIII and CIV after BN-PAGE was performed using established methods [6, 12]. For Complex I, III, and IV detection, Anti-NDUFB8 mouse monoclonal antibody (1:2500 dilution) (ab110242, Abcam), Anti-UQCRC2 rabbit monoclonal antibody 1:2500 dilution (ab203832, Abcam), and Anti-MTCO1 (rabbit monoclonal antibody (1:5000 dilution) (ab203912, Abcam) were used respectively. Secondary anti-rabbit antibody (for CIII and CIV, stabilized peroxidase conjugate, Thermo Scientific) and anti-mouse (for CI, stabilized peroxidase conjugate, Thermo Scientific) antibodies were used. SuperSignal Western Femto Sensitivity kit was used to develop the signal, which was captured using a Fluro-S Max multi imager [6].

Supplementary References

1. Nemkov T, Hansen KC, D'Alessandro A. A three-minute method for high-throughput quantitative metabolomics and quantitative tracing experiments of central carbon and nitrogen pathways. *Rapid communications in mass spectrometry : RCM* **31**, 663-673 (2017).

2. Clasquin MF, Melamud E, Rabinowitz JD. LC-MS data processing with MAVEN: a metabolomic analysis and visualization engine. *Current protocols in bioinformatics* **Chapter 14**, Unit14 11 (2012).

3. Xia J, Sinelnikov IV, Han B, Wishart DS. MetaboAnalyst 3.0--making metabolomics more meaningful. *Nucleic Acids Res* **43**, W251-257 (2015).

4. Chappell JB, Perry SV. Biochemical and osmotic properties of skeletal muscle mitochondria. *Nature* **173**, 1094-1095 (1954).

5. Seifert EL, Bezaire V, Estey C, Harper ME. Essential role for uncoupling protein-3 in mitochondrial adaptation to fasting but not in fatty acid oxidation or fatty acid anion export. *The Journal of biological chemistry* **283**, 25124-25131 (2008).

6. Mileykovskaya E*, et al.* Arrangement of the respiratory chain complexes in Saccharomyces cerevisiae supercomplex III2IV2 revealed by single particle cryo-electron microscopy. *The Journal of biological chemistry* **287**, 23095-23103 (2012).

7. Jha P, Wang X, Auwerx J. Analysis of Mitochondrial Respiratory Chain Supercomplexes Using Blue Native Polyacrylamide Gel Electrophoresis (BN-PAGE). *Current protocols in mouse biology* **6**, 1-14 (2016).

8. Wittig I, Braun HP, Schagger H. Blue native PAGE. *Nat Protoc* **1**, 418-428 (2006).

9. Williams EG*, et al.* Systems proteomics of liver mitochondria function. *Science* **352**, aad0189 (2016).

10. Frenzel M, Rommelspacher H, Sugawa MD, Dencher NA. Ageing alters the supramolecular architecture of OxPhos complexes in rat brain cortex. *Exp Gerontol* **45**, 563-572 (2010).

11. Nohara K*, et al.* Ammonia-lowering activities and carbamoyl phosphate synthetase 1 (Cps1) induction mechanism of a natural flavonoid. *Nutr Metab (Lond)* **12**, 23 (2015).

12. Bazan S*, et al.* Cardiolipin-dependent reconstitution of respiratory supercomplexes from purified Saccharomyces cerevisiae complexes III and IV. *The Journal of biological chemistry* **288**, 401-411 (2013).
