# Supplementary material for: Nobiletin fortifies mitochondrial respiration in skeletal muscle to promote healthy aging against metabolic challenge
Source: Nat Commun. 2019 Aug 28;10:3923. doi: 10.1038/s41467-019-11926-y (PMC6713763; doi:10.1038/s41467-019-11926-y)
Supplement: Supplementary file 2 — Reporting Summary [file 41467_2019_11926_MOESM2_ESM.pdf]

nature research

Corresponding author(s): Zheng Chen

Last updated by author(s): Jul 14, 2019

Reporting Summary

Nature Research wishes to improve the reproducibility of the work that we publish. This form provides structure for consistency and transparency in reporting. For further information on Nature Research policies, see Authors & Referees and the Editorial Policy Checklist.

Statistics

For all statistical analyses, confirm that the following items are present in the figure legend, table legend, main text, or Methods section.

n/a	Confirmed	
☐	☒	The exact sample size (n) for each experimental group/condition, given as a discrete number and unit of measurement
☐	☒	A statement on whether measurements were taken from distinct samples or whether the same sample was measured repeatedly
☐	☒	The statistical test(s) used AND whether they are one- or two-sided<br>*Only common tests should be described solely by name; describe more complex techniques in the Methods section.*
☒	☐	A description of all covariates tested
☒	☐	A description of any assumptions or corrections, such as tests of normality and adjustment for multiple comparisons
☒	☐	A full description of the statistical parameters including central tendency (e.g. means) or other basic estimates (e.g. regression coefficient) AND variation (e.g. standard deviation) or associated estimates of uncertainty (e.g. confidence intervals)
☒	☐	For null hypothesis testing, the test statistic (e.g. F, t, r) with confidence intervals, effect sizes, degrees of freedom and P value noted<br>*Give P values as exact values whenever suitable.*
☒	☐	For Bayesian analysis, information on the choice of priors and Markov chain Monte Carlo settings
☒	☐	For hierarchical and complex designs, identification of the appropriate level for tests and full reporting of outcomes
☒	☐	Estimates of effect sizes (e.g. Cohen's d, Pearson's r), indicating how they were calculated

Our web collection on statistics for biologists contains articles on many of the points above.

Software and code

Policy information about availability of computer code

Data collection	ActiView software (circadian behavior), PiezoSleep Mouse Behavioral Tracking System (sleep recording), Oxymax-CLAMS (metabolic chamber assays), Mass Matrix, MAVEN (metabolite analysis), Seahorse XF24 Analyzer (mitochondrial bioenergetics).
Data analysis	Tophat2 v2.0.12, Cufflinks v2.2.1 (RNA-seq); MetaboAnalyst 3.0 package, GENE-E (metabolite analysis); additional data were analyzed and plotted using Microsoft Excel, SigmaPlot and GraphPad Prism.

For manuscripts utilizing custom algorithms or software that are central to the research but not yet described in published literature, software must be made available to editors/reviewers. We strongly encourage code deposition in a community repository (e.g. GitHub). See the Nature Research guidelines for submitting code & software for further information.

Data

Policy information about availability of data

All manuscripts must include a data availability statement. This statement should provide the following information, where applicable:

- Accession codes, unique identifiers, or web links for publicly available datasets
- A list of figures that have associated raw data
- A description of any restrictions on data availability

Data that support the findings of the current study are available from the corresponding author upon reasonable request. The GEO accession ID for the RNA-seq dataset is GSE134304 [https://www.ncbi.nlm.nih.gov/geo/query/acc.cgi?acc=GSE134304]. The uncropped images of key western blots are presented in Supplementary Fig 7.

Field-specific reporting

Please select the one below that is the best fit for your research. If you are not sure, read the appropriate sections before making your selection.

☒ Life sciences ☐ Behavioural & social sciences ☐ Ecological, evolutionary & environmental sciences

For a reference copy of the document with all sections, see nature.com/documents/nr-reporting-summary-flat.pdf

Life sciences study design

All studies must disclose on these points even when the disclosure is negative.

Sample size	Sample size was determined by established procedures for particular experiments; power analysis was performed when needed.
Data exclusions	None
Replication	All experiments were replicated at least three times with biologically independent samples
Randomization	Aged mice acquired were randomized into treatment groups
Blinding	Lab members collaborated on most experiments, and blinding was performed among lab members whenever possible (e.g., mouse techs provide the mice without disclosing treatment group).

Reporting for specific materials, systems and methods

We require information from authors about some types of materials, experimental systems and methods used in many studies. Here, indicate whether each material, system or method listed is relevant to your study. If you are not sure if a list item applies to your research, read the appropriate section before selecting a response.

Materials & experimental systems

n/a	Involved in the study	
☐	☒	Antibodies
☐	☒	Eukaryotic cell lines
☒	☐	Palaeontology
☐	☒	Animals and other organisms
☒	☐	Human research participants
☒	☐	Clinical data

Methods

n/a	Involved in the study	
☒	☐	ChIP-seq
☒	☐	Flow cytometry
☒	☐	MRI-based neuroimaging

Antibodies

Antibodies used	BMAL1 (epitope: aa 1-230, Cocalico Biologicals) and IgY were affinity purified using the same protein, 1:4000), RORα (epitope: aa 105-122, Cocalico Biologicals, 1:1000), RORγ (epitope: aa 212-230, Cocalico Biologicals; 1:1000), REV-ERBα (Millipore; 1:1000) and GAPDH (Santa Cruz SC25778; 1:5000).<br>Anti-NDUFB8 mouse monoclonal antibody (1:2500 dilution) (ab110242, Abcam), Anti-UQCRC2 rabbit monoclonal antibody 1:2500 dilution (ab203832, Abcam), and Anti-MTCO1 (rabbit monoclonal antibody (1:5000 dilution) (ab203912, Abcam) were used respectively. Secondary anti-rabbit antibody (for CIII and CIV, stabilized peroxidase conjugate, Thermo Scientific) and anti-mouse (for CI, stabilized peroxidase conjugate, Thermo Scientific) antibodies were used.
Validation	All antibodies were validated following manufecturer's instruction.

Eukaryotic cell lines

Policy information about cell lines

Cell line source(s)	C2C12 from ATCC
Authentication	The vendor provided evidence of authentication, and secondary validation via established differentiation protocols
Mycoplasma contamination	Cells were tested for mycoplasma using commercial kits and confirmed negative
Commonly misidentified lines (See ICLAC register)	none

Animals and other organisms

Policy information about studies involving animals; ARRIVE guidelines recommended for reporting animal research

Laboratory animals	Mice at different ages
Wild animals	n/a
Field-collected samples	n/a
Ethics oversight	All animal studies were approved by UTHealth Center for Laboratory Animal Medicine and Care (CLAMC) and were conducted in compliance with CLAMC designated guidelines.

Note that full information on the approval of the study protocol must also be provided in the manuscript.
